# Marine Jellyfish Collagen and Other Bioactive Natural Compounds from the Sea, with Significant Potential for Wound Healing and Repair Materials

**DOI:** 10.3390/md23060252

**Published:** 2025-06-13

**Authors:** Ana-Maria Pesterau, Antoanela Popescu, Rodica Sirbu, Emin Cadar, Florica Busuricu, Ana-Maria Laura Dragan, Carolina Pascale, Ana-Maria Ionescu, Claudia Florina Bogdan-Andreescu, Marius-Daniel Radu, Cezar Laurentiu Tomescu

**Affiliations:** 1 Organizing Institution for Doctoral University Studies of “Carol Davila”, Faculty of Pharmacy, University of Medicine and Pharmacy Bucharest, Dionisie Lupu Street, No. 37, Sector 2, 020021 Bucharest, Romania; ana-maria.pesterau@drd.umfcd.ro (A.-M.P.); sirbu_27@yahoo.com (R.S.); ana-maria-laura.dragan@drd.umfcd.ro (A.-M.L.D.); carolina.pascale@drd.umfcd.ro (C.P.); 2 Faculty of Pharmacy, “Ovidius” University of Constanta, Capitan Aviator Al. Serbanescu Street, No. 6, Campus, Corp C, 900470 Constanta, Romania; antoniapopescu2002@yahoo.co.uk (A.P.); busuricuflori@yahoo.com (F.B.); 3 Faculty of Medicine, “Ovidius” University of Constanta, University Alley, No. 1, Campus, Building B, 9000470 Constanta, Romania; cezar.laurentiu@gmail.com; 4 Clinical Hospital C F Constanta, 1 Mai Blvd., No. 3–5, 900123 Constanta, Romania; 5 Faculty of Dental Medicine, Department of Speciality Disciplines, “Titu Maiorescu” University, 031593 Bucharest, Romania; claudia.andreescu@prof.utm.ro; 6 Faculty of Natural and Agriculture Science, “Ovidius” University of Constanta, University Alley, No. 1, Campus, Building B, 9000470 Constanta, Romania; marius.radu@univ-ovidius.ro; 7 “Sf. Ap. Andrei” County Clinical Emergency Hospital, Blvd. Tomis No. 145, 900591 Constanta, Romania

**Keywords:** marine bioactive compounds, marine metabolites, skin health, wound healing

## Abstract

Skin health must be ensured at all times in the case of wounds when the skin is subjected to traumatic actions that require multiple wound-healing measures. Wound healing is a complex, multi-phase biological process critical for restoring skin integrity after trauma. This study investigates the development and evaluation of a novel composite hydrogel formulated from collagen peptides extracted from the jellyfish *Rhizostoma pulmo* and hydroethanolic extracts from the brown alga *Cystoseira barbata*, both sourced from the Romanian Black Sea coast. Throughout the work, the characteristics due to the biochemical compositions of the extracts from the brown alga *C. barbata* and from the jellyfish *R. pulmo* are highlighted as important, emphasizing the content of polysaccharides, proteins, and lipids. Total phenol content was analyzed for three extracts from natural products. The biochemical composition, antioxidant, antimicrobial, and in vitro wound-healing properties of the components and their composite (JPC-ALG) were assessed. The rheological behavior and optical microscopy studies of collagen hydrogels were prepared. The general mechanisms of wound healing with the involvement of polysaccharides and collagen peptides existing in all categories of extracts were highlighted. The study of the effects of JPC-ALG composites and individual extracts on fibroblast and keratocyte cell lines is also presented. Results demonstrated that the composite exhibited synergistic effects, enhancing fibroblast and keratinocyte migration and proliferation, key factors in wound closure. The findings support the potential application of this marine-derived bioactive composite as a promising biomaterial for wound-healing therapies.

## 1. Introduction

Skin health is an important concern not only in routine cosmetic care but also in situations where the skin undergoes significant trauma, such as wounds requiring complex healing processes, as noted by Gu et al. (2024) and Childs et al. (2017) [1,2]. Continuous exposure to external and internal factors can compromise the health of the skin, the body’s primary interface with the environment, leading to disturbances as reported by Anderson et al. (2012) [3]. Currently, the maintenance of skin health and effective wound healing is crucial due to the high risk of morbidity associated with skin injuries. In this context, treatments based on natural biocompounds are being increasingly explored for their potential in promoting wound healing, as indicated by Han et al. (2017) and Reza Farahpour et al. (2019) [4,5].

Wound healing involves restoring the skin’s structure and function by repairing anatomical disruptions, which can sometimes extend beyond the skin to deeper tissues such as the subcutaneous layer, muscles, nerves, tendons, blood vessels, and even bones, as described by Frykberg et al. (2015) and Öztürk et al. (2011) [6,7]. Skin wounds can be categorized as acute or chronic. demonstrated that acute skin wounds typically undergo normal healing phases and recover within approximately four weeks [8]. On the contrary, chronic wounds heal slowly and deviate from the standard phases of wound healing, often becoming highly susceptible to infection, as shown by Sen et al. (2019), Shilo et al. (2013), and Kiritsi et al. (2017) [9,10,11]. Chronic wounds, including arterial ulcers, venous ulcers, diabetic ulcers, and pressure ulcers, are frequently associated with patient immobility, advanced age, impaired blood circulation, and systemic diseases, as reported by Velnar et al. (2009), Schreml et al. (2010), and Beldon et al. (2010) [12,13,14]. Effective management of chronic wounds necessitates not only local wound care but also the identification and treatment of the underlying etiological factors, as emphasized by Hochstein et al. (2014) [15]. The treatment of chronic wounds and restoration of skin integrity present significant clinical challenges, often requiring multidisciplinary teams and considerable healthcare resources to achieve favorable outcomes, as demonstrated by Frykberg et al. (2015) [6]. Conventional therapeutic approaches, primarily involving analgesics and antibiotics for pain relief and infection prophylaxis, remain the cornerstone of chronic wound management, as noted by Schreml et al. (2010) [13]. However, in recent years, natural biomaterials such as collagen have been increasingly incorporated into wound dressings to enhance healing by providing a protective barrier and maintaining optimal moisture levels, as shown by Hochstein et al. (2014) and Felician et al. (2019) [15,16].

Collagen has been recommended as a wound dressing material in various formulations, including powders, gels, pastes, and collagen-impregnated dressings, according to Chattopadhyay et al. (2014) and Zhang et al. (2020) [17,18]. Beyond collagen, other natural materials have been identified as beneficial for maintaining skin health, as reported by Sirbu et al. (2019) and Cadar et al. (2023) [19,20]. Bioactive collagen-derived peptides, depending on their amino acid composition, exhibit diverse biological activities, including immunomodulatory effects (Fan et al., 2013), angiotensin-converting enzyme (ACE) inhibitory properties (Barzideh et al. (2014)), antibacterial activity (Ennaas et al. (2015); Ennaas et al. (2016)), and antioxidant capacity (Cadar et al. (2024)) [21,22,23,24,25]. Moreover, Asserin et al. (2015) demonstrated that collagen peptides, unlike native collagen, are readily digested by gastric enzymes, absorbed via peptide transporters, and distributed systemically, thus facilitating targeted biological activity [26]. Banerjee et al. (2015) demonstrated that collagen peptides in the skin act as false collagen-degrading peptides, sending deceptive signals to fibroblasts to stimulate the synthesis of new collagen fibers. Additionally, these peptides exhibit chemotactic properties and promote cell migration and proliferation, thereby actively participating in the wound-healing process [27]. Traditionally, collagen used for medical applications has been extracted from terrestrial animals, particularly cattle and pigs. However, collagen derived from terrestrial sources carries significant risks, including the potential transmission of diseases such as transmissible spongiform encephalopathy (TSE), bovine spongiform encephalopathy (BSE), foot-and-mouth disease (FMD), and various viral pathogens, all of which can represent a threat to human health [28]. In response to these concerns, marine organisms have been explored as alternative collagen sources. Jellyfish, notably *Rhopilema esculentum*, have garnered significant attention due to their nutritional and pharmacological properties, as highlighted by Addad et al. (2011), Yu et al. (2014), and Khong et al. (2016) [29,30,31]. The antioxidant properties of proteins extracted from *R. esculentum* were demonstrated by Zhuang et al. (2010), while their hemostatic effects were reported by Cheng et al. (2017) [32,33]. Felician et al. (2019) further described the applications of collagen derived from *R. esculentum* in wound healing [16]. Another jellyfish species, *Rhizostoma pulmo*, has similarly attracted interest as a source of collagen and collagen-derived peptides. Research conducted by James et al. (2023), D’Ambra et al. (2015), and De Domenico et al. (2019) has explored the extraction and characterization of collagen from *R. pulmo* [34,35,36]. Moreover, Cadar et al. (2023) reported encouraging findings on the potential applications of jellyfish-derived collagens in promoting wound healing [37].

Beyond jellyfish, marine algae also represent a valuable source of biomaterials for wound-healing applications. Rich in polysaccharides, algae possess numerous biocompounds with beneficial effects on the wound-healing process. The antioxidant activities of marine algae have been well documented by Wang et al. (2019, 2020), Jayawardena et al. (2020), Yalçın et al. (2021), and Cadar et al. (2023) [38,39,40,41,42]. Furthermore, the multiple health benefits of seaweeds, including antioxidant, anti-inflammatory, and regenerative properties, have been emphasized by Cadar et al. (2025) [43]. In particular, the anti-inflammatory effects of fucoidan, a sulfated polysaccharide extracted from brown algae, have been evidenced by Liyanage et al. (2023) and Jayasinghe et al. (2023) [44,45].

Effective wound management remains a major challenge in clinical practice due to the complexity of the skin’s repair process following injury. Wound healing involves a coordinated cascade of events aimed at restoring skin structure and function, often disrupted by trauma, chronic disease, or infection. Traditional treatments frequently rely on synthetic agents, but there is growing interest in bioactive natural compounds that support and enhance tissue regeneration. In this study, we explore the wound-healing potential of a novel hydrogel composite developed from marine-derived collagen peptides (*Rhizostoma pulmo*) and polysaccharide-rich algal extracts (*Cystoseira barbata*).

This study aims to characterize the physicochemical properties of a novel pharmaceutical formulation derived from marine-origin materials. Specifically, it focuses on collagen extracted from the jellyfish *Rhizostoma pulmo* and hydroalcoholic extracts from the brown seaweed *Cystoseira barbata*, both collected from the Black Sea. We propose to investigate the individual and combined effects of jellyfish collagen and seaweed extracts using a variety of physicochemical techniques, with the goal of evaluating their potential as alternatives to conventional pharmaceutical formulations for wound-healing therapies. The primary objective of this study was to demonstrate the physicochemical, microbiological, antioxidant, and regenerative properties of novel marine-origin biocompounds—specifically, collagen extracted from the jellyfish *Rhizostoma pulmo* from the Black Sea, in synergy with the biological and pharmacological properties of biocompounds from hydroalcoholic extracts obtained from the brown alga *Cystoseira barbata*. A secondary aim was to highlight the potential of these marine-derived components, jellyfish collagen, and biocompounds derived from brown algae as natural resources with superior qualities compared to conventional biomaterials of terrestrial animal or synthetic origin, particularly in applications related to wound healing and overall skin health.

To this end, we aim to investigate key compounds known to support tissue regeneration, including proteins, polysaccharides, and polyphenolic compounds.

For both extracts, we will first establish their proximate composition. In the case of the jellyfish collagen extract, we will further analyze the collagen type through SDS-PAGE, circular dichroism (CD) spectroscopy, Fourier-transform infrared (FT-IR) spectroscopy, and amino acid composition profiling. Additionally, we will assess the total and individual polyphenolic content of both materials.

We propose to evaluate the antioxidant and antimicrobial activities of the biocompounds present in each extract, both individually and in the newly developed composite formulation, as these biological activities are known to facilitate wound healing. The potential synergistic effects of these compounds on critical phases of the wound repair process will be investigated through physicochemical and microbiological assays.

As an initial step, we will assess the wound-healing potential of the new composite formulation in vitro using the cell culture scratch assay. This will include tests on BALB/3T3 clone A31 murine embryonic fibroblast monolayers and HaCaT human keratinocyte cell lines.

## 2. Results

Pharmaceutical formulations for skin tissue regeneration in wound treatment are made with marine extracts based on jellyfish collagen from *Rhizostoma pulmo* and marine algae extract from *Cystoseira barbata*. The two basic materials originate from the Black Sea and are analyzed first individually and then in pharmaceutical formulations destined for skin tissue restoration in wound healing. Obtaining the extracts from the raw materials collected from the Black Sea littoral led to the following results.

### 2.1. Chemical Characteristics for Ingredients

#### Proximate Composition Data of Collagen Peptide Extract and Hydroalcoholic Algal Extract

Collagen hydrolysate from the jellyfish *R. pulmo* was extracted using two methods: acid-soluble collagen (ASC) and pepsin-soluble collagen (PSC), yielding 43.2% and 47.5%, respectively. Subsequent enzymatic hydrolysis produced collagen peptides in white powder form, with dry weight yields of 52.3% (ASC) and 59.5% (PSC). The proximate composition of these collagen peptides, along with the ethanolic extract of *C. barbata*, a brown alga collected from the Black Sea, is detailed in Table 1. Protein, lipid, and carbohydrate contents in *R. pulmo* were expressed as percentages of dry weight (DW) across different anatomical parts of the jellyfish: whole body (W), bell (B), oral arms (OA), and gonads (G). For comparative purposes, the literature data on *R. pulmo* compositions are also presented [46,47,48]. Collagen hydrolysate appeared as a white gelatinous substance and was subsequently incorporated into composites prepared with *C. barbata* extracts. In *R. pulmo*, protein (primarily collagen) was the predominant component, followed by lipids (4.9 ± 0.81% in the whole body), and minimal carbohydrate content (0.59 ± 1.25% in the whole body). For *C. barbata*, brown algae from the Black Sea, and proximate compositions from hydroethanolic extracts were analyzed and compared with findings by Cadar et al. (2019) [49]. Additionally, Pesterau et al. (2024) reported a validated protocol for extracting collagen from *R. pulmo* jellyfish harvested along the Romanian Black Sea coast [48].

### 2.2. Jellyfish R. pulmo Physico—Date for the Collagen Structure

#### 2.2.1. SDS-PAGE Analysis

**Figure 1:**
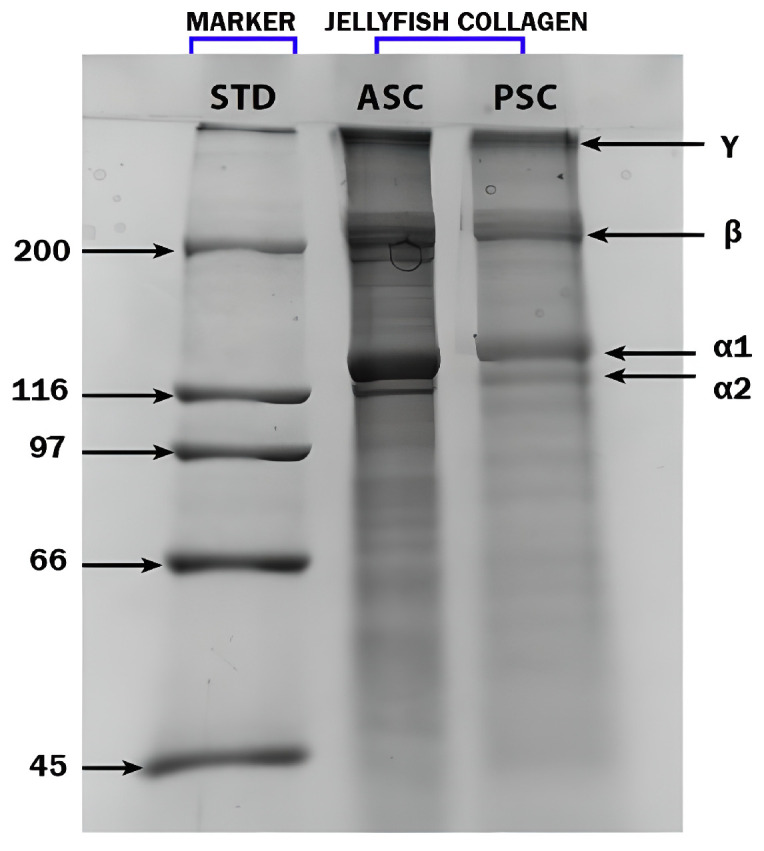
Result of SDS-PAGE analysis of *R. pulmo* collagen.

To analyze the structural characteristics of jellyfish collagen extracted from *R. pulmo*, SDS-PAGE analysis was performed, as shown in Figure 1. Both collagen extracts obtained using the acid-soluble collagen (ASC) and pepsin-soluble collagen (PSC) methods exhibited similar electrophoretic profiles. The presence of two distinct α chains—α1 and α2—confirms the identity of the extracted collagen as type I collagen. This profile was benchmarked against a bovine type I collagen standard used as a molecular weight marker. The SDS-PAGE results revealed distinct protein bands in the range of 110–150 kDa corresponding to the α chains, with additional β- and γ-chain bands observed above 200 kDa, indicating dimeric and trimeric cross-linked forms, respectively. These findings align with previous studies by Addad et al. (2011), James et al. (2023), De Domenico et al. (2019), Widdowson et al. (2018), and Paradiso et al. (2019), further validating the classification of *R. pulmo* collagen as structurally and functionally comparable to vertebrate type I collagen [28,29,34,36,50].

#### 2.2.2. Circular Dichroism Spectral Analyses

**Figure 2:**
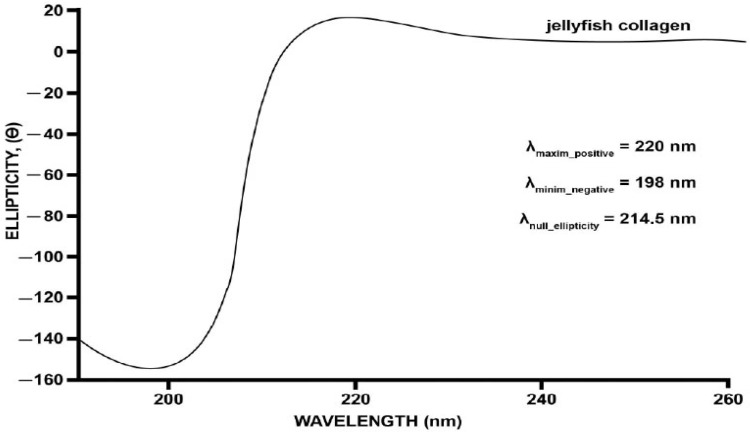
Circular dichroism spectrum of *R. pulmo* collagen.

To characterize the secondary structure of *R. pulmo* collagen, circular dichroism (CD) spectroscopy was performed, with the resulting spectra presented in Figure 2. The CD spectrum of jellyfish-derived collagen exhibited a positive ellipticity peak at approximately 220 nm and a negative minimum at 198 nm. These spectral features are indicative of a preserved triple-helical structure, which is characteristic of native collagen.

#### 2.2.3. FT-IR Analysis

**Figure 3:**
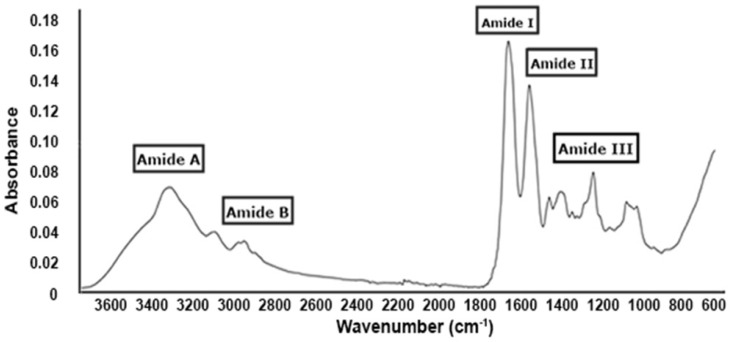
FT-IR spectra for collagen extracted from the jellyfish *R. pulmo*.

The FT-IR spectrum of collagen extracted from *R. pulmo*, as shown in Figure 3, displays characteristic absorption bands confirming the presence and integrity of type I collagen.

A broad band at 3283 cm^−1^ corresponds to amide A, attributed to N–H stretching vibrations involved in hydrogen bonding. The band at 2934 cm^−1^ (amide B) arises from the asymmetric stretching of CH_2_ groups. Prominent absorption peaks at 1647 cm^−1^, 1534 cm^−1^, and 1241 cm^−1^ correspond to amide I, II, and III, respectively. The amide I band is primarily due to C=O stretching vibrations coupled with COO^−^ and hydrogen bonding, whereas the amide II band results from CN stretching and NH bending. The amide III band is attributed to C–O stretching and NH deformation coupled with CN stretching.

Additional peaks at 1444 cm^−1^ and 1334 cm^−1^ are associated with C–N stretching of cyclic proline and CH_2_ vibrations in the proline side chains, respectively. The presence of these peaks—particularly the amide II band in the 1533–1537 cm^−1^ range—confirms the retention of the triple-helical structure, consistent with the findings reported by James et al. (2023) [34]. Overall, the FT-IR analysis supports the identification of *R. pulmo* collagen as type I collagen with a preserved triple helix conformation. These FT-IR spectral results support the presence of a stable triple-helix structure for type I collagen, consistent with previous studies by Barzideh et al. (2014) on collagen from the jellyfish *Chrysaora* sp. [22].

#### 2.2.4. Amino Acid Composition

The amino acid composition of *R. pulmo* harvested from the Romanian Black Sea coast is presented in Table 2. For comparison, reference values reported by James et al. (2023) for *R. pulmo* collected from the Goa coast, India, are also included [34]. The profiles are largely comparable, with glycine (*Gly*) being the most abundant amino acid in both samples, followed by glutamic acid (*Glu*). Notably, tryptophan (*Trp*) was detected in *R. pulmo* from both the Black Sea and the Goa coast. In contrast, cystine (*Cys*), histidine (*His*), and serine (*Ser*) were present in relatively low quantities in the Black Sea specimen.

James et al. (2023) identified a typical amino acid profile in *R. pulmo*, including *Gly*, *Glu*, alanine (*Ala*), aspartic acid (*Asp*), and leucine (*Leu*) [34]. Our analysis of the Black Sea *R. pulmo* revealed a similar but slightly varied sequence: *Gly* > *Glu* > *Leu* > *Ala* > *Asp* > lysine (*Lys*) > arginine (*Arg*). Furthermore, Leone et al. (2015) reported the presence of all essential amino acids (EAAs)—*His*, isoleucine (*Ile*), *Leu*, *Lys*, methionine (*Met*), phenylalanine (*Phe*), threonine (*Thr*), and valine (*Val*)—in *R. pulmo* from the Mediterranean Sea, although *Trp* was not identified in their sample [46].

### 2.3. Polyphenols Content

#### 2.3.1. Total Phenols Content and Total Flavonoid Content

The total phenolic content of *R. pulmo* jellyfish collagen hydrolysate determined by us was 1540 ± 291 µg GAE/g DW, a result comparable to those in other literature results [46]. For *C. barbata* algae hydroalcoholic extract, the total phenolic content was 60 ± 0.42 mg GAE/g DW. These results obtained by us are comparable with the results reported by Mhadhebi et al. (2014), (50.3 mg GAE/g DW) and Generalić Mekinić et al. (2019) (48.09 mg GAE/g DW) [51,52]. Manev et al. (2013) and Kosanić et al. (2015) also reported data on the total phenolic content existing in brown algae of the genus *Cystoseira* [53,54]. The total flavonoid content determined by us in the hydroethanolic extracts of *C. barbata* was 58.5 ± 0.42 mg QE/g DW. The result is comparable with the value of 55.14 ± 1.078 mg QE/g DW reported by Cadar (2018) [55]. These compounds support the antioxidant activity of *R.pulmo* jellyfish extracts. Our data, although lower, also attests to the possibility of antioxidant activity of *R. pulmo* extracts. However, the brown alga *C. barbata* possesses much higher quantities of both total phenolic and flavonoid compounds. The total content of phenolic compounds for *C. barbata* from the Black Sea was also reported by Cadar et al. (2019) and Dragan et al. (2023) [49,56]. Studies on the content of phenolic compounds were also reported by Cadar et al. (2019) and Dragan et al. (2023) [49,56].

#### 2.3.2. Individual Phenolic Acids

The individual phenolic acids identified in hydroalcoholic extracts of *C. barbata* and in extract of *R. pulmo* are presented in Table 3.

Polyphenols identified in the collagen peptide extract of *R. pulmo*. HPLC-DAD analysis revealed that vanillic acid is the most abundant, followed by benzoic acid and feluric acid. P-hydroxybenzoic acid and caffeic acid are found in moderate amounts. Compared with the composition of the brown seaweed *C. barbata* (with an analyzed polyphenol content of 309.22 mg w.w.), the jellyfish collagen extract from *R. pulmo* showed a much lower content of individual polyphenols (6.58 mg w.w.). Only gallic acid, caftaric acid, and syringic acid were identified in the extracts from *R. pulmo*.

### 2.4. Physico-Chemical Characteristics for the New Wound-Healing Preparations

The new composites have in their composition collagen hydrolysates (collagen peptides) from *R. pulmo* and hydroalcoholic extracts from the brown alga *C. barbata*.

#### 2.4.1. Organoleptic Characteristics of the New Preparations Obtained

The organoleptic characteristics of the composites obtained from collagen peptide hydrolysates and *C. barbata* brown algae extracts are presented in Table 4. We note that the color of the preparations varies depending on the percentage of brown algae extract, ranging from yellowish white to brownish white.

Different morphologic forms of *R. pulmo*-derived collagen peptides were obtained. The lyophilized form shows a white, fluffy texture. In *R. pulmo* and collagen peptide films, the preparation is presented as a porous membrane structure, which is relevant for biomedical applications, such as wound dressings. The hydrogel formulation was obtained by combining collagen peptides with a 5%, 10%, and 15% hydroalcoholic extract of *C. barbata* (brown seaweed). In addition, collagen films incorporating hydroalcoholic extracts of brown seaweed in different concentrations—5%, 10%, and 15%—demonstrate the potential of modulating the characteristics of the composite by the extract concentration.

#### 2.4.2. Rheological Study of JPC-ALG Composite Hydrogels

**Figure 4:**
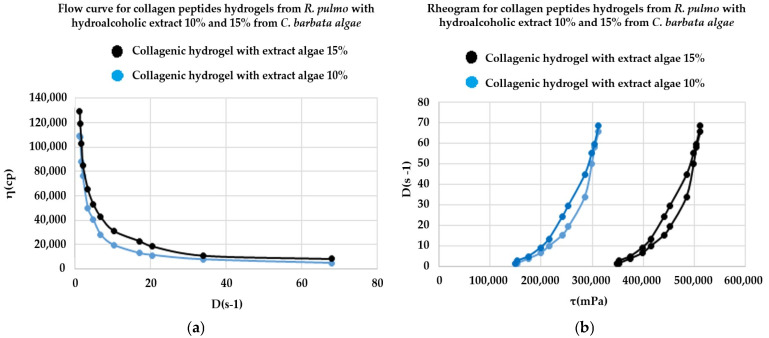
(**a**) Flow curve of collagen preparations with *C. barbata* brown alga extract. (**b**) Rheogram for the preparation with brown seaweed extract *C. barbata*.

The rheological parameters obtained allowed for the construction of the rheological profiles of the formulations. Figure 4a,b illustrate the flow curves and corresponding rheograms for two preparations containing collagen peptides combined with differing concentrations (10%) of *C. barbata* hydroethanolic extract (JPC-ALG). The rheological behavior of the *R. pulmo* collagen peptide-based hydrogels, formulated with various concentrations of the brown algae extract, exhibited a consistent pseudoplastic (shear-thinning) behavior across the entire range of shear rates applied. Both the flow curves and rheograms (Figure 4a,b) demonstrate uniform behavior, and the presence of hysteresis loops in the rheograms confirms thixotropic characteristics under increasing and decreasing shear rates. Similar rheological characteristics for collagen-based preparations have previously been described by Cadar et al. (2019) and Cherim et al. (2019) [57,58].

#### 2.4.3. Microscopic Study of JPC-ALG Composites Intended for Wound Healing

**Figure 5:**
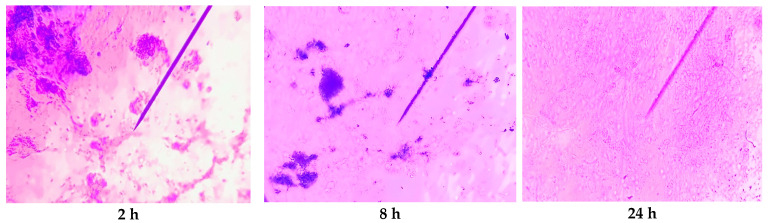
Photomicrographs showing the evolution in the homogeneity of the JPC-ALG composite.

JPC-ALG hydrogels were prepared by combining collagen peptides derived from *R. pulmo* with hydroalcoholic extracts of the brown alga *Cystoseira barbata*. Figure 5 presents microstructural images of the JPC-ALG composite captured at 2, 8, and 24 h following the mixing process. The progressive development of the composite structure over time was clearly observed. Notably, after 24 h, a homogeneous and stable composition was achieved, indicating complete integration and uniform dispersion of the bioactive compounds from both marine sources.

### 2.5. Antioxidant Activity

Antioxidant activity was tested by two methods: the DPPH test and the reducing power assay. These methods are different in principle but both attest to the antioxidant capacity of both the individual components and the new preparations in the form of collagen peptide hydrogels with different algae compositions.

#### 2.5.1. DPPH Test

**Figure 6:**
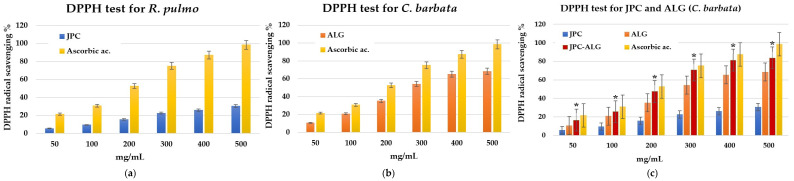
Results for DPPH radical scavenging activity of marine-derived compounds ((**a**)—JPC, collagen peptides extracted from *R. pulmo*; (**b**)—ALG, *C. barbata* extract) and (**c**)—newly formulated JPC-ALG composite, compared to the standard antioxidant, ascorbic acid. Antioxidant activity was measured by the ability of each sample to scavenge DPPH radicals. Results are expressed as mean ± SD (*n* = 3). * *p* < 0.05 for JPC-ALG compared to JPC.

The evolution of DPPH radical scavenging activity at different concentrations is depicted in Figure 6. Figure 6a,b illustrate the DPPH radical scavenging activity for the individual biocompounds: collagen peptides (JPC) from *R. pulmo* at varying concentrations (Figure 6a) and hydroalcoholic extracts from the brown alga *Cystoseira barbata* (ALG) (Figure 6b).

Figure 6c shows the variation in DPPH radical scavenging activity for different concentrations of the newly formulated JPC-ALG composite. It is evident that the antioxidant activity of the new composite is enhanced compared to the individual components. This increase in antioxidant activity can be attributed to several factors, notably the high content of polysaccharides in *C. barbata* seaweed extract (ALG), which complements the relatively lower polysaccharide content in the collagen-based JPC extract from *R. pulmo*. Furthermore, the total phenolic compounds (TPCs) present in the *C. barbata* extract contribute significantly to the overall antioxidant effect, with additional polyphenols found in the JPC from *R. pulmo*. The IC_50_ value for the collagen peptides from *R. pulmo* was 816.45, and for the hydroalcoholic extracts of *C. barbata*, it was 275.2. The IC_50_ value for the JPC-ALG composite was 210.50, and for ascorbic acid, which serves as the standard, it was 189.90. Based on the IC_50_ values obtained from the DPPH test on the initial bioproducts extracted from jellyfish (*R. pulmo*) and brown algae (*C. barbata*), as well as on the JPC-ALG preparation, it is evident that the antioxidant activity of hydroalcoholic extracts from *C. barbata* is more potent than that of the biocompounds from *R. pulmo*. In comparison, the new JPC-ALG composite of marine origin demonstrates the highest antioxidant activity. Various antioxidant activities were compared to ascorbic acid, which served as the standard. To evaluate statistically significant differences between the compounds analyzed in the DPPH test, one-way ANOVA analysis was applied. The result indicates a significant difference between the groups analyzed with the new JPC-ALG formulation compared to the JPC formulation at *p* < 0.05, noted in the graph in Figure 6c with the symbol *.

#### 2.5.2. Antioxidant Activity by Reducing Power

**Figure 7:**
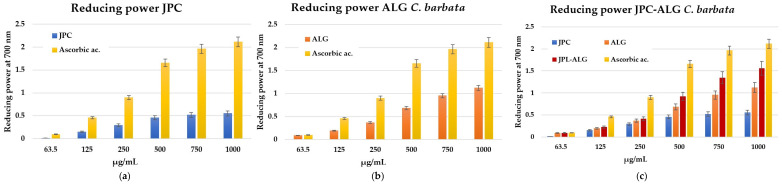
Results for reducing power assay of marine-derived compounds. (**a**)—JPC, collagen peptides extracted from *R. pulmo*; (**b**)—ALG, *C. barbata* extract, (**c**)—newly formulated JPC-ALG composite, compared to the standard antioxidant, ascorbic acid. Results are presented as mean ± SD (*n* = 3).

The same trend is observed in the reducing power assay, where it is evident that the antioxidant activity increases in the new preparation created by mixing biocompounds from *R. pulmo* and the brown alga *C. barbata*. The reducing power indicates that antioxidant compounds act as electron donors, capable of reducing oxidized intermediates in the lipid peroxidation process. In this way, antioxidants can function as both primary and secondary antioxidants [59]. The reducing power was concentration-dependent and increased with higher concentrations, see Figure 7a–c. As shown in Figure 7c, the reduction potency of the new preparation is higher than that of the collagen peptides (JPC) from *R. pulmo* and the *C. barbata* algal extracts. Previous studies by Mhadhebi et al. (2014), Kosanić et al. (2015), and Sirbu et al. (2019) have demonstrated that antioxidant activity is largely supported by polyphenol compositions [51,54,60].

### 2.6. Antimicrobial Activity

**Figure 8:**
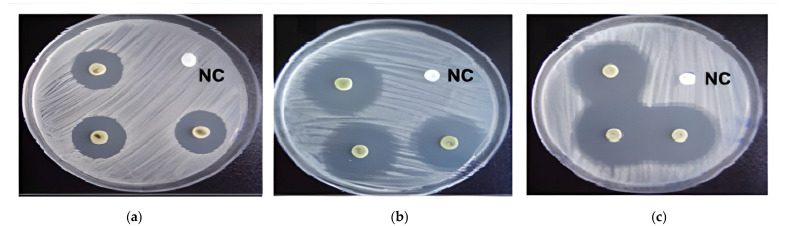
(**a**) Antimicrobial activity of JPC extract from *R. pulmo* against *S. aureus*; NC-negative control. (**b**) Antimicrobial activity of 10%, 15%, and 20% *C. barbata* brown alga extracts (ALG) against *S. aureus*; NC—negative control. (**c**) Antimicrobial activity of the preparation JPC-ALG (collagen with *C. barbata* alga 10%, 15%, and 20%) against *S. aureus*; NC—negative control.

Biocompounds from marine resources exhibit notable antimicrobial activity. The results of antibacterial activities, measured as zones of inhibition, are expressed in millimeters. These zones were observed for disks filled with JPC extracts from *R. pulmo*, ALG ethanolic extracts from the brown alga *Cystoseira barbata*, and the new composites made from JPC-ALG at varying algal concentrations. The tested bacterial strains included four Gram-negative species: *Klebsiella pneumoniae* (ATCC 13883), *Pseudomonas aeruginosa* (ATCC 27853), *Proteus mirabilis* (ATCC 25933), and *Escherichia coli* (ATCC 25322), as well as two Gram-positive species: *Staphylococcus aureus* (ATCC 25923) and *Streptococcus epidermidis* (ATCC 12228). Figure 8a–c display the antimicrobial activity against *S. aureus* using the respective biocompounds. *S. aureus* strains were found to be the most sensitive to the tested extracts. Other pathogens exhibited varying responses to the action of the *C. barbata* ALG ethanolic extracts and *R. pulmo* JPC extracts. The seaweed extract solutions used were at concentrations of 10%, 15%, and 20%, with 85% ethanol serving as a negative control. As shown in Figure 8a, JPC extracts demonstrated antimicrobial activity against *S. aureus*. Figure 8b shows the antimicrobial effect of different concentrations of *C. barbata* brown alga extracts (10%, 15%, and 20% ethanol), which exhibited stronger activity than the JPC collagenic extracts from *R. pulmo*. Finally, Figure 8c illustrates the antimicrobial activity of the composite mixtures (JPC-ALG) with varying concentrations of *C. barbata* hydroalcoholic extracts.

**Figure 9:**
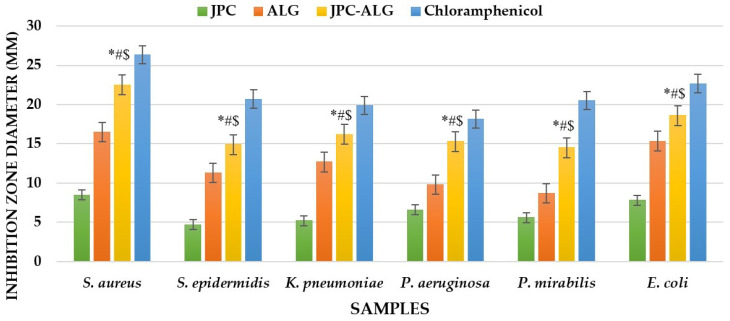
The antimicrobial activities of JPC from *R. pulmo*, the extract from *C. barbata*, and the new pharmaceutical composite JPC-ALG. Chloramphenicol served as a control. Data are expressed as the mean ± SD of three replicates (*n* = 3). * *p* < 0.05 significantly different from JPC; # *p* < 0.05 significantly different from ALG; $ *p* < 0.05 significantly different from Chloramphenicol.

A comparison of the three figures clearly indicates that the new JPC-ALG composite exhibits greater antimicrobial activity than the individual extracts. This enhanced effect is likely due to the synergistic action of its biochemically rich composition, including polyphenols and polysaccharides, rich components from *C. barbata*, and protein compounds from *R. pulmo*. Figure 9 demonstrates that the studied biocompounds—JPC from *R. pulmo* and ALG from *C. barbata*—exhibited antimicrobial activity against both Gram-negative and Gram-positive bacteria, with the highest sensitivity observed against *Staphylococcus aureus*.

The seaweed extract solution was used at a concentration of 15%, while chloramphenicol (5 mg/mL) served as the positive control. Among the tested strains, *Proteus mirabilis* showed the lowest susceptibility. Minimum inhibitory concentration (MIC) values, as presented in Table 5, ranged from 25 µg/mL to >100 µg/mL.

*Proteus mirabilis* exhibited the lowest sensitivity to all tested extracts, highlighting variability in bacterial susceptibility. Notably, the antimicrobial activity of the composites appears to be strongly influenced by the presence of *C. barbata* algal extracts.

### 2.7. Biological Evaluation of New Composite with JPC-ALG for Wound-Healing Application

**Figure 10:**
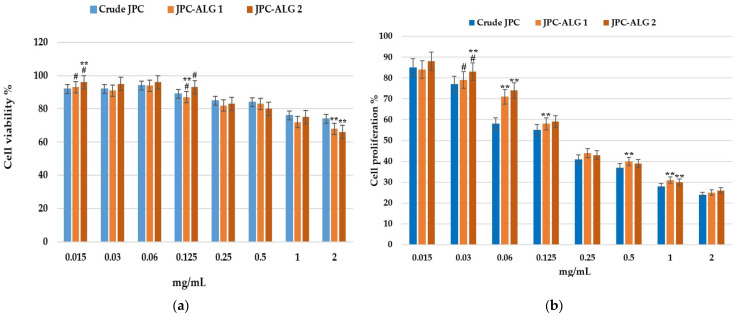
Cytocompatibility screening performed on BALB/3T3 cell line clone A31, exposed to JPC (collagen peptides from *R. pulmo*), JPC–ALG1 (JPC with 10% extract of *C. barbata*), and JPC–ALG2 (JPC with 20% extract of *C. barbata*), in the 0.015–2 mg/mL concentration range: (**a**) Cell viability after 24 h of incubation; (**b**) Cell proliferation after 48 h of incubation. Values are expressed as the mean ± standard deviation (SD) of eight replicates (*n* = 8) from two independent experiments. Statistical significance (*p* < 0.01) is indicated as follows: ** *p* < 0.01 significantly different of JPC-ALG 1 or JPC-ALG 2 from JPC; # *p* < 0.01 significantly different of JPC–ALG 2 from JPC and from JPC-ALG 1.

Preliminary biological investigations were conducted using BALB/3T3 clone A31 fibroblasts, in accordance with the ISO-10993-1 (Geneva, Switzerland, 2018). Standard, due to the critical role of these fibroblasts in the wound-healing process [61,62]. To determine the optimal concentration for performing the scratch assay, cytotoxicity assessments were first carried out. These tests evaluated the effects of JPC (collagen peptides from *R. pulmo*), as well as the composite formulations JPC-ALG1 (collagen peptides with 10% *C. barbata* extract) and JPC-ALG2 (collagen peptides with 20% *C. barbata* extract). The cytotoxicity results, which guided the concentration selection for the scratch assay, are presented in Figure 10.

As shown in Figure 10, cell viability decreased with increasing sample concentrations across all tested formulations. After 24 h of incubation, high cell viability was maintained at lower concentrations (0.015–0.125 mg/mL), with values exceeding 87% to 96% for all tested samples. As concentrations increased (2 mg/mL), a gradual decline in viability was observed, yet values remained around 66–74% after 24 h of exposure (Figure 10a). At 48 h, a further reduction in cell proliferation was recorded with increasing concentrations (Figure 10b). Specifically, cell proliferation remained above 80% at 0.01 mg/mL for all samples but declined to 37–40% at 0.5 mg/mL, and to 24–26% at 2 mg/mL. These results indicate acceptable cytocompatibility at lower concentrations, consistent with previous findings on jellyfish-derived collagen peptides (JSP), as reported by Migone et al. (2022) and Li et al. (2017) [63,64]. Statistical analysis revealed significant differences (*p* < 0.01) between the analyzed groups only at certain concentrations. At 0.015 mg/mL, the JPC-ALG2 (**, #) group exhibited significantly different cell viability from JPC and from JPC-ALG1 (#), while JPC-ALG1 was significantly different only from JPC-ALG2 (**, #). At a concentration of 125 mg/mL, the JPC-ALG1 (**, #) formulations showed statistical differences from JPC and JPC-ALG2 (#). JPC-ALG2 (#) showed a statistically significant difference only from JPC-ALG1 (**, #). At a high concentration of 2 mg/mL, the JPC-ALG1 (**) and JPC-ALG2 (**) formulations showed statistically significant differences from JPC (**). A steady decrease with increasing concentration was observed in cell proliferation. Statistical differences were evident at low and medium concentrations. Thus, at a concentration of 0.03 mg/mL, JPC-ALG2 (**, #) showed a statistically significant difference from JPC and JPC-ALG1, while JPC-ALG1(#) differed only from JPC-ALG2 (**, #). At a concentration of 0.06 mg/mL, formulations with JPC-ALG1 (**) and JPC-ALG2 (**) showed statistical differences from JPC, and at a concentration of 0.125 mg/mL, only JPC-ALG1 (**) was statistically different from JPC. At higher concentrations, at 0.5 mg/mL and 1 mg/mL, JPC-ALG1 (**) showed statistically significant differences only from JPC, and at the concentration of 1 mg/mL and JPC-ALG2 (**) formulation, as well as JPC-ALG1 (**), showed statistically significant differences from JPC. At the same time, these results confirm good cytocompatibility at low concentrations and also show that JPC-ALG1 and JPC-ALG2 formulations positively impact cell viability and proliferation at low doses, which confer their regenerative properties.

#### 2.7.1. Wound Healing by the Fibroblast Scratch Test

**Figure 11:**
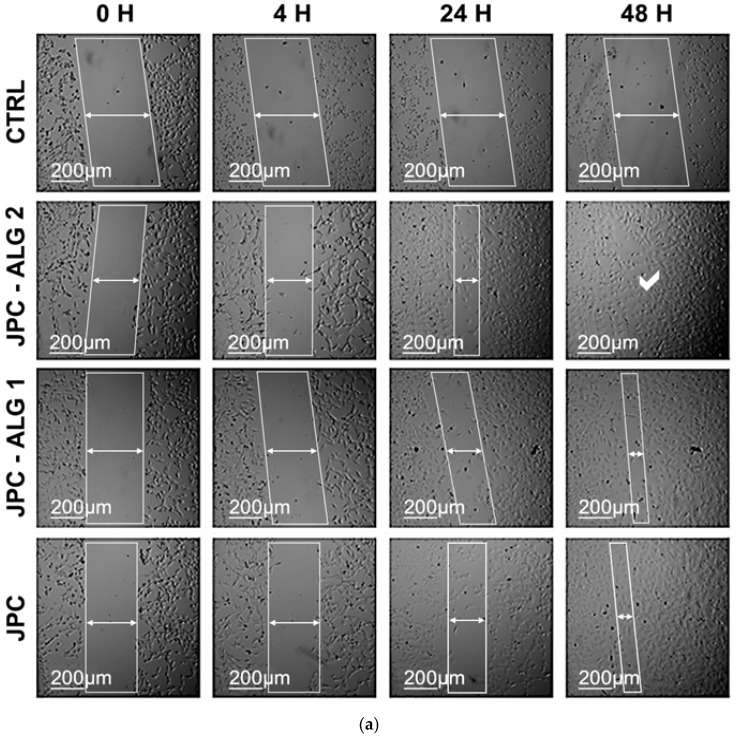

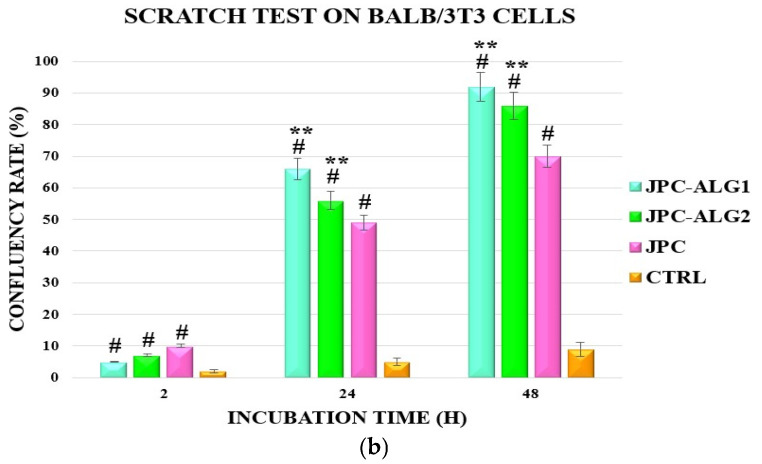
(**a**) Scratch test on BALB/3T3 clone A31 murine embryonic fibroblast cell monolayers treated with *R. pulmo* extract and *C. barbata* extract (JPC, JPC-ALG 1, and JPC-ALG 2. Control consists of untreated cells (CONTROL). (**b**) Evolution of confluency rate calculated as a percentage (%) in the scratch assay performed on BALB/3T3 fibroblast cell line with JPC-ALG1, JPC-ALG2, and JPC treatments, compared with untreated control (CTRL). The assay was monitored at 2, 24, and 48 h. Data are expressed as mean ± SD (*n* = 8). Statistical differences were determined by one-way ANOVA analysis as follows: ** *p* < 0.01 statistically significant difference compared to JPC; # *p* < 0.01 statistically significant difference compared to CTRL.

The scratch test is a well-established in vitro method used to mimic wound healing by mechanically creating a gap in a confluent monolayer of cells. This assay models the migration and proliferation of cells toward the wound site, as demonstrated by Migone et al. (2022) and Morgner et al. (2022) [63,65]. In this study, a standardized scratch was made using a pipette tip to simulate tissue injury. To minimize the influence of cell proliferation and focus on migratory behavior, the assay was conducted in a medium containing low serum (1% FBS), in accordance with the recommendations by Grada et al. (2017) [66]. The effects of JPC, JPC-ALG 4, and JPC-ALG 2 on fibroblast migration were evaluated by monitoring wound closure at 4, 24, and 48 h post-injury (Figure 11a).

All tested samples significantly accelerated cell migration and enhanced the confluence rate compared to the untreated control (Figure 11b). At 48 h, the control group exhibited only 5% confluence, whereas cells treated with JPC reached 66% scratch closure. Notably, JPC-ALG 1 and JPC-ALG 2 demonstrated even higher rates of wound closure, achieving approximately 86–92% closure.

At 24 h and 48 h, the symbol ** showed a significant difference for JPC-ALG1and JPC-ALG2, respectively, versus JPC, and the #—symbol showed a significant difference between JPC-ALG1, JPC-ALG2, and JPC versus CTRL for *p* < 0.01. These findings support the potential of different concentrations of JPC-ALG1 and JPC-ALG2 formulations in skin tissue regeneration.

#### 2.7.2. Wound Healing by Keratocyte Scratch Assay

**Figure 12:**
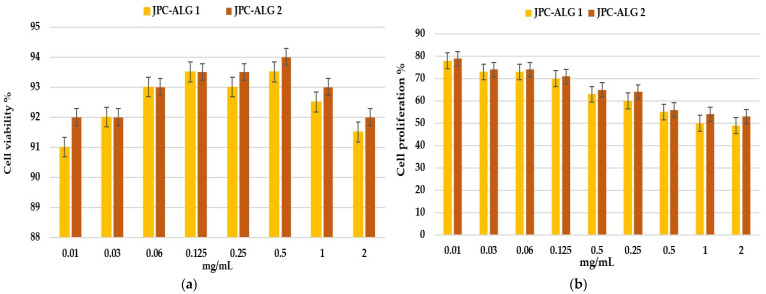
Cytotoxicity screening performed on the HaCat cell line, exposed JPC-ALG1 and JPC-ALG 2 in the 0.015–2 mg/mL concentration range: (**a**) cell viability after 24 h of incubation; (**b**) cell proliferation after 48 h of incubation. The values indicated in the figure are means ± SD; *n* = 8.

A preliminary cytotoxicity assessment of JPC, JPC-ALG 1, and JPC-ALG 2 on HaCaT keratinocyte cells was performed (Figure 12a,b). After 24 h of exposure, none of the samples exhibited cytotoxic effects. However, after 48 h, a concentration-dependent reduction in cell proliferation was observed. Specifically, after 24 h at 0.125 mg/mL, cell viability remained increased to 93.5%, while at higher concentrations (1–2 mg/mL), proliferation decreased to 91.5–94%. As shown in Figure 12b, after 48 h treatment with JPC-ALG 1 and JPC-ALG 2 at a concentration of 0.125 mg/mL, cell proliferation levels reached 70–71%, while higher concentrations (1–2 mg/mL) led to a reduction in proliferation, with values ranging from 49% to 54%.

**Figure 13:**
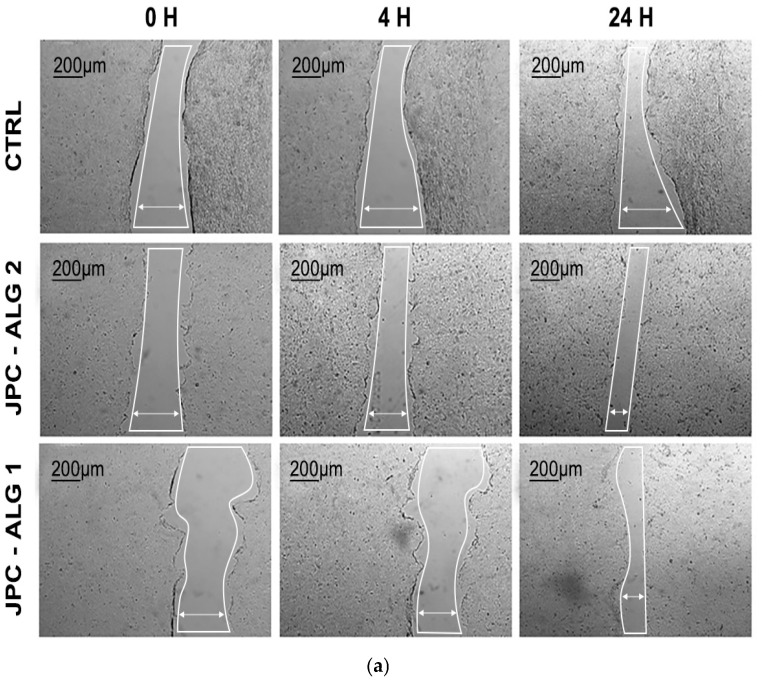

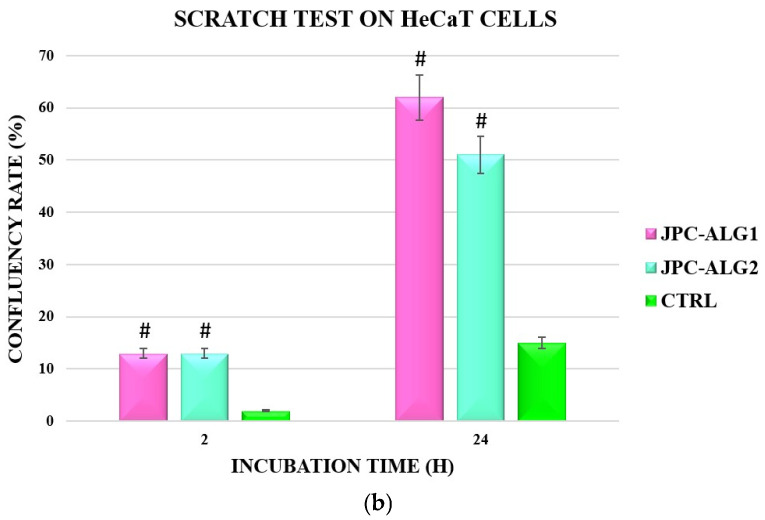
(**a**) Representative microfilms (4× size) of treated and control (untreated) HaCat cell monolayers. (**b**) Confluency rate (%) for scratch assay performed on the HaCaT cell line, treated with the new formulations JPC–ALG1 and JPC–ALG2, compared to the untreated control (CTRL), at incubation times of 2 h and 24 h. Data obtained are expressed as mean ± standard deviation (*n* = 8). Statistical analysis was performed using one-way ANOVA: (*p* < 0.01) and symbol (#) indicate statistically significant differences compared to the control group (CTRL).

These results confirm the good biocompatibility of both JPC-ALG 1 and JPC-ALG 2 formulations in HaCaT human keratinocyte cell lines. Figure 13a,b illustrate the results of the scratch assay performed on HaCaT cell monolayers treated with the composite preparations containing collagen peptides from *R. pulmo* and hydroethanolic extracts from *C. barbata* at concentrations of 10% (JPC-ALG 1) and 20% (JPC-ALG 2), respectively. Untreated cells were used as control. The observed wound closure and cell proliferation data support the potential of these marine-derived composites in wound-healing applications. Compared to the control group, which exhibited a 10% confluency rate, treatment with JPC-ALG 1 and JPC-ALG 2 composites resulted in significantly enhanced cell migration, with confluency rates of 51% and 62%, respectively, as shown in Figure 12b. The scratch assay was employed to evaluate the effect of the composite formulations on HaCaT keratinocyte migration. Using a single-chamber analysis, the wound closure capacity was monitored over a 24 h period. Figure 13a,b illustrate the increased migratory response induced by JPC-ALG 1 and JPC-ALG 2, demonstrating their potential efficacy in promoting wound healing through enhanced keratinocyte motility.

The scratch assay showed a different behavior depending on the treatment administered (JPC-ALG1 or JPC-ALG2) and the incubation time. Statistical analysis was performed using one-way ANOVA (*p* < 0.01). At both 2 h and 24 h of incubation, both analyzed groups JPC-ALG1 and JPC-ALG2 showed statistically significant differences from the control (CTRL) marked with the symbol (#).

This biocompatibility was also noticed in other jellyfish collagen fractions by Felician et al. (2019) and Migone et al. (2022) [16,63].

## 3. Discussions

The four phases of normal wound healing can be distinct or overlapping. The first phase, hemostasis, begins immediately after tissue injury. During this phase, platelet aggregation leads to the release of various chemotactic factors, including platelet-derived growth factors: PDGF, TGF-β1, and TGF-β2, which recruit inflammatory cells such as neutrophils, macrophages, and leukocytes to the wound site, thereby providing protection against infection. Platelets also contribute to clot formation, while fibrin, beyond forming the clot matrix, plays a protective role by limiting microbial proliferation and preserving tissue integrity. Following hemostasis, the inflammatory phase ensues. This phase is characterized by the activation of neutrophils and the establishment of a robust inflammatory response. Neutrophils amplify the action of pro-inflammatory cytokines and secrete antimicrobial substances, including reactive oxygen species cationic peptides, (ROS), and proteases, as demonstrated by Shaw et al. (2009) and Eming et al. (2007) [67,68]. Neutrophils, key effector leukocytes in wound healing, are rapidly activated and transmigrate across endothelial barriers within 1–2 h post-injury. Their recruitment is mediated by pro-inflammatory cytokines, such as TNF-α, IL-1β, and IFN-γ, as demonstrated by Gonzalez et al. (2016) and Wang et al. (2018) [69,70]. The third phase, the proliferative phase, typically spans from 4 to 21 days post-injury. During this phase, critical events such as angiogenesis, extracellular matrix (ECM) deposition, and re-epithelialization occur. The ECM formation is initiated by platelet disaggregation stimulated by PDGF, leading to the production of proteoglycans and collagen, as demonstrated by Li et al. (2007) and Olczyk et al. (2014) [71,72]. In addition to epidermal and dermal cells, inflammatory cells secrete numerous mediators that regulate and stimulate the proliferation and migration of smooth muscle cells, fibroblasts, and keratinocytes within the wound. In response to hypoxia, angiogenic factors, such as factors: VEGF, PDGF, and basic fibroblast growth factor b-FGF, are important in the emergence of new blood vessels, as demonstrated by Demidova-Rice et al. (2012) [73]. As the proliferative phase progresses, extracellular matrix (ECM) components such as fibronectin, glycosaminoglycans, proteoglycans, thrombospondins, tenascin, vitronectin, and various collagens are synthesized and deposited, as reported by Barroso et al. (2019) [74]. Angiogenesis, the formation of new blood vessels, is a key event during this phase, driven by the release of factors like TGF-β, PDGF, and FGF from platelets. These factors not only stimulate the formation of new blood vessels but also facilitate the repair of damaged ones, as reported by Singh et al. (2017) [75]. Demidova-Rice et al. (2012) highlighted the essential role of these factors in promoting vascularization of the wound site [73]. Martin et al. (2015) identified the appearance of granulation tissue, which is highly vascularized and serves as a scaffold for further tissue formation [76]. Additionally, TGF-β is a critical growth factor that promotes tissue repair by supporting cellular functions, including fibroblast proliferation, as reported by Okur et al. (2020) [77]. As the epithelial cells migrate toward the center, they meet, halting migration and initiating the formation of the basement membrane, as reported by Mangoni et al. (2016) [78]. The formation of the basement membrane is a key marker that signals the completion of the epithelialization process. The remodeling phase, which follows proliferation, can last from two to three weeks to over a year, depending on the severity of the injury [79]. Over time, as the wound matures, the granulation tissue undergoes remodeling, and a scar is formed, primarily composed of collagen I, with fewer and less mature blood vessels, as reported by Li et al. (2007) and by Okur et al. (2020) [71,77]. Growth factors with polypeptide structures, such as those produced by inflammatory cells and fibroblasts, play an essential role in remodeling by stimulating cell proliferation and driving tissue regeneration [37,78]. While some wounds heal following the normal sequence of phases, others may deviate from this pattern due to the intervention of various external and internal factors, leading to delayed or incomplete healing. Collagen peptides derived from *Rhizostoma pulmo* (*R. pulmo*) have shown remarkable potential in wound healing by promoting the production of chemotactic factors and fibroblasts, as demonstrated in this study. Similar findings were reported by Felician et al. (2019), who showed that collagen peptides from *Rhopilema esculentum* jellyfish improved the remodeling phase of wound healing by evaluating key factors such as TGF-β1 and b-FGF in the skin of laboratory mice [16]. The use of collagen from marine sources in wound healing has also been explored by Wang et al. (2015) and Hu et al. (2017) [80,81]. In 2020, Okamura et al. introduced a new composite biomaterial made from moon jellyfish collagen and porcine type I collagen, which significantly accelerated the wound-healing process when used as a graft (artificial dermis) [82]. The following year, Sumiyoshi et al. (2021) demonstrated that the external application of jellyfish collagen solution not only accelerated physiological wound healing but also prevented excessive scar genesis [83]. They highlighted that the property of jellyfish collagen to denature and degrade rapidly at body temperature makes it an ideal candidate for topical application. This characteristic also minimizes the risk of unexpected immune reactions that might arise from unidentified components of invertebrate collagen [83]. Additional data on the extraction, characteristics, and applications of *R. pulmo* jellyfish collagen have been provided by Pesterau et al. (2022, 2023, 2024) [84,85,86]. Their studies confirmed that the collagen extracted from *R. pulmo* possesses a type I collagen structure, as verified by SDS-PAGE and FT-IR. Circular dichroism spectral analyses are consistent with previous findings reported by Gopinath et al. (2014) and Barros et al. (2015), confirming the structural integrity of the collagen extracted from *R. pulmo* [87,88,89]. The collagen peptides from *R. pulmo* sourced from the Black Sea are rich in amino acids, and their composition was compared with that of *R. pulmo* jellyfish from other regions, including the Goa coast of India and the Mediterranean Sea. The extracts from *R. pulmo* contain protein compounds (60.48 ± 1.72%), with collagen making up 57.1 ± 0.6% of the total protein content. In contrast, polysaccharides are present in much smaller quantities (0.59 ± 1.25% W and 0.25 ± 0.65% G). When combined with protein extracts from *C. barbata* algae (20.98 ± 0.65%), the overall composition of the new PCL-ALG composite benefits from a synergistic blend of biocompounds. This includes both proteins and polyphenols from *R. pulmo*, as well as specific mono- and polysaccharides from *C. barbata*.

It is noteworthy that the biochemical composition of *C. barbata* may vary depending on geographic location and environmental conditions. The carbohydrate content in both fresh and dried *R. pulmo* tissue has been less consistently reported in the literature, with notable variations across studies, as reviewed by Cadar et al. (2023) [37]. As reported by Manev et al. (2013), protein, lipid, carbohydrate, and polyphenol contents may vary across regions of the Bulgarian Black Sea coast, including Shabla, St. Atanas, Rusalka, and Tsarevo [53]. It is also important to highlight the findings of Dragan et al. (2023), who reported detailed data on the bioactive compounds present in *C. barbata* collected from the Black Sea. Their work established correlations between the structural characteristics of major polysaccharides—fucoidan, laminarins, alginic acid, and alginates—and their suitability for wound treatment applications [56]. The critical role of polysaccharides in wound healing has also been substantiated by several studies, including those by Shen et al. (2021), Yuan et al. (2022), and Migone et al. (2022) [63,90,91].

The polysaccharide composition of *C. barbata* (60.25 ± 1.56%) further complements the collagen extracts from *R. pulmo*, providing a diverse range of beneficial compounds for wound healing. From our analysis of the biocompounds in the studied marine materials, we emphasize the high protein content in the hydrogel extracted from *R. pulmo* jellyfish and the high carbohydrate content predominantly polysaccharides in *C. barbata*.

Polysaccharides are biopolymers with diverse structural architectures, composed of either homopolysaccharide or heteropolysaccharide chains, arranged in linear or branched configurations [43]. Fucoidan, a sulfated polysaccharide characteristic of brown algae, has shown a particularly beneficial effect on wound healing, as discussed by Cadar et al. (2025) [43]. Conversely, proteinaceous compounds, especially collagen from *R. pulmo*, have been extensively studied for their therapeutic applications. Riccio et al. (2022) reported that jellyfish-derived collagens exhibit antiproliferative effects in various skin disorders, while Nudelman et al. (2019) proposed the use of jellyfish biomaterials as smart wound-dressing devices due to their bioactivity and biocompatibility [92,93].

Thus, the new PCL-ALG composite combines the therapeutic properties of collagen peptides, polyphenols, and polysaccharides from both *R. pulmo* and *C. barbata*, creating a highly promising biocomposite for wound-healing applications. The types of polysaccharides found in brown algae, particularly fucoidan, are well-known for their role in suppressing skin lesions, as reported by Wang et al. (2024) [94]. Migone et al. (2022) also demonstrated the beneficial impact of polysaccharides in wound healing [63]. The JPC-ALG composite, in the form of a hydrogel, was studied for its rheological behavior to assess its stability.

Rheological analyses produced flow curves and rheograms for both the individual components and the JPC-ALG composite. The results revealed that the new composite behaves as a non-Newtonian fluid, characteristic of the Ostwald de Waele rheological model, which ensures its stability over time. It was observed that the formulated hydrogels retained their structural and rheological properties over time, displaying a flow behavior that conforms to the Ostwald–de Waele (power law) rheological model [55]. This model describes the non-Newtonian, shear-thinning behavior characteristic of the formulations. Comparable rheological studies on collagen-based hydrogels and algal extracts have been reported by Sîrbu et al. (2010), further supporting the findings of this work [95].

Optical microscopy also showed that the composite achieves excellent homogeneity after 24 h.

Antioxidant activity. The composition of the JPC-ALG composite supports its antioxidant activity, as demonstrated by specific tests like DPPH and reducing power assays. These activities are higher in the composite than in the individual JPC and ALG components at the same concentrations. Reducing oxidative stress in wounds is essential, and composites with strong antioxidant activity are beneficial for this purpose. The role of antioxidants in wound healing has been well documented, as shown by De Domenico et al. (2019) and Migone et al. (2022) [36,63]. The antioxidant properties of collagen from *R. pulmo* have been confirmed by several studies, including those by James et al. (2023), Leone et al. (2015), and Gao et al. (2024) [34,46,96]. Additionally, Diao et al. (2021) demonstrated that natural compounds can help mitigate oxidative stress and protect the skin [97]. The antioxidant activity of *C. barbata* is also notable, with research by Cadar et al. (2019) and Trifan et al. (2019) calling attention to the antioxidant potential of its algal biocompounds [57,98]. Cadar et al. (2019) explored the correlation between these two methods to assess antioxidant activity in the hydroalcoholic extract of *C. barbata* [57]. Furthermore, they found significant correlations between total phenolic content (TPC) and antioxidant activity (IC_50_ values), confirmed by Pearson correlation coefficient analysis conducted by Trifan et al. (2019) [98]. Yegdaneh et al. (2016) also reported the antioxidant activity of biocompounds from *Cystoseira* species, which are rich in tannins, saponins, sterols, and triterpenes, among the most abundant constituents in these species [99].

The antioxidant properties are primarily attributed to the polyphenolic compounds in marine extracts. *C. barbata* contains a high concentration of total phenols (60 ± 0.42 mg GAE/g DW) and flavonoid compounds (58.5 ± 0.42 mg QE/g DW). In contrast, extracts from *R. pulmo* have a significantly lower total phenol content (1540 ± 291 µg/g DW). We notice that data for alcoholic extracts of *R pulmo* in ethanol have also been reported by Leone et al. (2015) (2079.3 ± 302 µg GAE/g DW), who studied *R. pulmo* jellyfish alongside other jellyfish harvested from the Mediterranean Sea [46]. While 14 individual polyphenols were identified in *C. barbata*, only 3 were identified in *R. pulmo*. The antioxidant activity of the JPC-ALG composite was assessed using the DPPH method at concentrations of 400 mg/mL, resulting in a value of 81.3 ± 0.4 mg/mL. This was higher than the antioxidant activity of ALG (*C. barbata* extracts) at 65.3 ± 0.25 mg/mL and JPC (*R. pulmo* extracts) at 25.9 ± 0.6 mg/mL. In the reducing power assay, the JPC-ALG composite at 750 µg/mL exhibited a value of 1.345 ± 0.1 µg/mL, compared to 0.952 ± 0.3 µg/mL for ALG and 0.521 ± 0.2 µg/mL for JPC.

Antimicrobial activity. Edwards et al. (2004) accentuate that wounds are vulnerable to contamination by various microorganisms, which can either obstruct or support the healing process [100]. For effective wound healing, it is important to differentiate between wound contamination, colonization, and infection, as discussed by Okur et al. (2020) and Bowler et al. (2001) [77,101]. In our study, the highest antimicrobial activity was observed against *S. aureus*, with the JPC-ALG composite producing a zone of inhibition of 22.5 ± 0.2 mm. This was larger than the zones observed for JPC (8.5 ± 0.1 mm) and ALG (16.5 ± 0.2 mm). The second most sensitive bacterium was *E. coli*, with a zone of inhibition of 18.6 ± 0.1 mm for JPC-ALG, compared to 7.8 ± 0.1 mm for JPC and 15.33 ± 0.1 mm for ALG. Against *K. pneumoniae*, the zone of inhibition for JPC-ALG was 16.2 ± 0.1 mm, compared to 5.2 ± 0.1 mm for JPC and 12.7 ± 0.1 mm for ALG. For *P. aeruginosa*, the JPC-ALG composite produced a zone of inhibition of 15.3 ± 0.1 mm, compared to 6.6 ± 0.2 mm for JPC and 9.8 ± 0.2 mm for ALG. The antimicrobial activity against *P. mirabilis* was also higher for JPC-ALG (14.5 ± 0.1 mm) compared to JPC (5.6 ± 0.2 mm) and ALG (8.7 ± 0.25 mm), and against *S. epidermidis*, JPC-ALG (14.9 ± 0.1 mm) showed a larger zone of inhibition than JPC (4.7 ± 0.1 mm) and ALG (11.3 ± 0.2 mm). Table 5 presents the minimum inhibitory concentrations (MIC) for the green algae extracts. The most sensitive strains were *S. aureus* and *E. coli*, with MIC values of 25 µg/mL. The strains *S. epidermidis*, *P. aeruginosa*, and *K. pneumoniae* exhibited MIC values of 50 µg/mL, while *P. mirabilis* showed the least sensitivity, with an MIC value of up to 100 µg/mL. The antimicrobial properties of the preparations used in wound healing are crucial, as demonstrated by Ovington et al. (2003) and Simões et al. (2018) [102,103]. The observed differences in antimicrobial activity between bacterial strains can be attributed to structural and biochemical differences in their cell envelopes. For instance, *S. aureus*, a Gram-positive bacterium with a thick peptidoglycan layer, demonstrated high sensitivity to both *C. barbata* and JPC-ALG composites. This is likely due to the increased permeability of Gram-positive membranes to bioactive polyphenols and peptides, which can disrupt membrane integrity and interfere with protein synthesis. In contrast, *Proteus mirabilis* and *Klebsiella pneumoniae*, Gram-negative strains, possess an outer membrane rich in lipopolysaccharides that act as a barrier to many antimicrobial agents, which may explain their lower susceptibility. The enhanced effect observed in the composite (JPC-ALG) can be attributed to the synergistic interaction between collagen-derived peptides and phenolic compounds, improving penetration and disruption mechanisms against bacterial cells. Previous studies have similarly reported the antimicrobial potential of brown algae. For instance, Ozdemir et al. (2006) investigated brown algae from the coast of Izmir, Turkey; Taskin et al. (2007) studied samples from the Aegean Sea; and Alghazeer et al. (2013) reported minimum inhibitory concentrations (MICs) for brown macroalgae from the western coast of Libya [104,105,106]. Kosanić et al. found that *C. barbata* extracts exhibit stronger antibacterial than antifungal activity [54]. Furthermore, Heijenoort (2001) emphasized that differences in microbial sensitivity are largely due to variations in cell wall structure and permeability among Gram-positive bacteria, Gram-negative bacteria, and fungi [107]. Additional antimicrobial studies on *C. barbata* from the Moroccan coast were reported by Ibtissam et al. (2009) [108].

From the scratch assays on BALB/3T3 fibroblast cells as well as on HaCat human keratocyte cells, the confluency rate was found to be increased compared to the control; the confluency rate was calculated as the percentage of scratch healing in relation to the initial scratch surface. Statistically significant differences were observed for BALB/3T3 cells at certain concentrations (** *p* < 0.01), showing that the extract obtained from brown seaweed is beneficial on cellular behavior, especially in the case of JPC-ALG2 formulation, where greater stimulation of cell viability and proliferation was observed compared to JPC and JPC-ALG1. We can also consider this effect to be dose-dependent and a possible synergistic mechanism between collagen peptides in jellyfish extract and bioactive compounds in brown algae extract.

In the HaCaT cell line, the results showed no statistically significant differences (** *p* < 0.01). In the evolution of confluency, the rate calculated in both a scratch assay performed on BALB/3Tr and on HaCaT keratocyte cells identified statistically significant differences in the new formulations compared to JPC and CTRL, respectively, at *p* < 0.01.

The trend observed in the cell viability and proliferation assays suggests a dose-dependent response of both fibroblasts and keratinocytes to the tested preparations. At lower concentrations (0.015–0.125 mg/mL), high cell viability and proliferation were maintained, indicating that the composite provides a biocompatible microenvironment supportive of cell growth. However, at higher concentrations (>1 mg/mL), a decrease in proliferation was noted, which may be attributed to increased osmotic stress or bioactive overload, warranting further optimization. The significant improvement in wound closure observed between 24 and 48 h suggests that the composite actively stimulates fibroblast migration and proliferation, critical steps during the proliferative phase of wound healing. These effects may be driven by enhanced production of extracellular matrix components (EMC), including collagen, as well as the stimulation of growth factors and chemotactic signals induced by the combined bioactive peptides and polysaccharides in the composite. In particular, sulfated polysaccharides, such as fucoidan from *C. barbata*, are known to interact with fibroblast growth factors, promoting angiogenesis and tissue remodeling, thus supporting the accelerated closure observed in vitro. Similarly, keratinocyte migration and proliferation were significantly enhanced by the JPC-ALG composites, indicating potential benefits for re-epithelialization in wound healing. The combined antioxidant and antimicrobial environment provided by the composite likely supports keratinocyte viability and function, reducing oxidative stress and infection risks. These cellular responses align with known mechanisms by which marine-derived collagen peptides and algal polysaccharides stimulate key signaling pathways involved in epidermal regeneration.

## 4. Materials and Methods

The jellyfish *R. pulmo* and the brown seaweed *C. barbata* were selected as the primary raw materials for the development of novel marine-derived composite pharmaceutical formulations intended for wound-healing applications. Both species were harvested from the marine waters along the Romanian Black Sea coast. Specimens of *R. pulmo* were collected from marine habitats in the vicinity of Mamaia Beach (coordinates: 44°14′28.20″ N, 28°37′13.19″ E) and the Constanța harbor area (coordinates: 44°10′24″ N, 28°38′18″ E). Samples of *C. barbata* were similarly collected from the Mamaia Beach region (coordinates: 44°14′28.20″ N, 28°37′13.19″ E) and from the southern sector of the Romanian coast, specifically the Costinești area (coordinates: 43°56′50″ N, 28°37′47″ E).

### 4.1. Chemical Reagents

All reagents were of analytical quality. The reagents were specific to the working phases of each technique and operational working method. *The reagents* used were specific to each phase of the applied techniques and methods. Glacial acetic acid (99–100%) was obtained from Merck KGaA (Darmstadt, Germany). SDS-PAGE reagents and molecular mass markers were sourced from Bio-Rad Laboratories (Hercules, CA, USA). Additionally, all other reagents and standards, including Type I collagen from bovine (used as a reference), were purchased from Sigma-Aldrich (Darmstadt, Germany). All reagents used were of analytical reagent grade. Controls used were purchased separately for each type of analysis.

### 4.2. Obtaining Extracts from Marine Resources

#### 4.2.1. Obtaining Collagen Extracts *R. pulmo*

*R. pulmo* jellyfish specimens harvested from the Black Sea were subjected to a pretreatment process involving successive cleaning operations. Initially, tissues were washed with sodium chloride (NaCl) solutions and ultrapure water, followed by treatment with a 0.5 M ethylene diamine tetraacetic acid sodium salt (EDTA-Na) solution for demineralization. Collagen extraction was subsequently performed according to the protocol described by Cadar et al. (2024) for the isolation of collagen from marine invertebrates [25]. Specifically, the jellyfish body components (arms and umbrella) were thoroughly washed and treated with a 0.5 M acetic acid solution for 30 min. Subsequently, 10% pepsin (*w*/*v*) was added, and the mixture was exposed to continuous agitation for three days at 4 °C. After enzymatic digestion, the suspension was centrifuged at 10,000 rpm for 1 h. The resulting supernatant was collected, and collagen was reprecipitated by the addition of a 2 M NaCl solution in the presence of 0.05 M Tris buffer (pH 7.0). The precipitate was isolated by centrifugation at 20,000 rpm for 1 h and then dissolved in 0.5 M acetic acid. The resulting solution underwent dialysis against ultrapure water for 2 h to remove residual acetic acid. All processing steps were conducted at 4 °C to preserve collagen integrity. The extracted collagen was subsequently either used directly as a hydrogel or subjected to lyophilization, depending on the intended application. The collagen yield (%) was calculated using the following Formula (1):

(1) $$\;\;\;\;\;\;\;\;Collagen\;yield\;\left(wet\right)\;\;\%=\frac{Weight\;of\;extracted\;collagen\;(g)}{weight\;of\;wet\;jellyfish\;(g)}\times 100$$

#### 4.2.2. Obtaining Collagen Peptides

To obtain collagen peptides, the method described by Felician et al. (2019) for the enzymatic hydrolysis of jellyfish collagen was employed [16]. Specifically, 1 g of collagen extracted from *Rhizostoma pulmo* was suspended in 200 mL of ultrapure water and incubated in a water bath at 37 °C. Collagenase type II was then added at a concentration of 5% (*w*/*w*, enzyme/substrate), and the mixture was continuously stirred for 5 h. To terminate the enzymatic reaction, the mixture was heated at 95 °C for 10 min. The suspension was then cooled to room temperature, and centrifuged at 5000 rpm for 30 min. The resulting supernatant, containing the jellyfish-derived collagen peptides (JPC), was collected. The peptides were either used immediately or lyophilized and stored at 4 °C for further applications.

#### 4.2.3. Extraction of *C. barbata* Extracts

The brown alga *C. barbata* is a large brown seaweed species, typically reaching lengths of 1.5–2 m, and forms a perennial group on rocky substrates along the Black Sea coast [49]. Cadar et al. (2017) reported detailed studies on the distribution of *C. barbata* habitats and the presence of important bioactive compounds within this species [109]. Fresh algal samples were collected and transported to the laboratory in new plastic bags filled with seawater to minimize evaporation. Upon arrival, the samples were rinsed thoroughly, first with fresh water and then with bidistilled water, to remove adhering debris and salts. The cleaned seaweed was subsequently shade-dried, crushed, and ground using an electric mixer. The resulting powder was sieved through a 0.3 mm mesh to obtain a uniform particle size. Extraction was performed using 70% and 90% (*v*/*v*) hydroalcoholic ethanol solutions. The powdered seaweed was soaked in the solvent at a solid-to-liquid ratio of 1:10 (*w*/*v*) for 24 h at room temperature (24 °C), using dark brown glass vessels sealed tightly to prevent exposure to light, air, and alcohol evaporation. A typical extraction involved a 1:4 weight ratio (20 g of seaweed to 100 g of solvent mixture). The mixtures were intermittently stirred to enhance the diffusion rate of bioactive compounds and to reduce extraction time. After extraction, the solutions were concentrated using a rotary evaporator to reduce their volume and subsequently filtered three times through Whatman No. 1 filter paper to obtain a clear, brown extract. The final extracts were collected in borosilicate glass containers and stored under refrigeration until further use [57].

### 4.3. Preparation of New Composite Preparations Based on Collagen Peptides from R. pulmo and Brown Alga C. barbata (JPC-ALG)

In the preparation of the new formulations, the methods described by Sirbu et al. (2019) and Bechir et al. (2014) for combining fish-derived collagen with various seaweeds were adapted, with modifications suitable for applications in skin tissue and oral mucosa treatments [60,110]. To obtain the new composite preparation, the hydrolyzed collagen peptide (HCP) extract from *R. pulmo* was first redissolved in 30 mL of bidistilled water. The pH of the solution was adjusted to 9 using either 1 M NaOH or 1 M HCl. Subsequently, the *C. barbata* algal extract was added at a mass ratio of 3:1 (collagen peptide: algal extract). The resulting mixture was stirred using a magnetic stirrer at 50 °C for 30 min to ensure homogeneity. After stirring, for 15 min the mixture was centrifuged at 10,000 rpm. The precipitate obtained was collected and divided into two portions: one portion was lyophilized for 48 h, while the other was preserved as a collagen peptide hydrogel combined with brown seaweed extract and stored under refrigeration for further use.

### 4.4. Determination of the Biochemical Compositions of Biocompounds from R. pulmo and C. barbata

The moisture content (%) of the samples was determined by drying 2 g of material in a temperature-controlled incubator at 105 °C until a constant weight was achieved. The ash content was measured by incinerating the samples in an electric oven at 500 °C for 4 h. All measurements were performed according to standardized AOAC procedures [111].

#### 4.4.1. The Biochemical Composition of Collagen Extracts from *Rhizostoma pulmo*

The methods were assessed using methods adapted from De Domenico et al. (2019), Leone et al. (2015), D’Ambra et al. (2022), and Migone et al. (2022) [36,46,47,63]. The total protein content was determined following the method described by Bradford (1976) [112]. Experimental procedures were adapted from De Domenico et al. (2019), utilizing 96-well round-bottom microplates and an Infinite M200 Quad4 Monochromator Detection System (Tecan, Männedorf, Switzerland) [36]. Bovine serum albumin (BSA) was used as the standard for calibration. All measurements are presented as the average of three independent determinations. Previous studies by De Domenico et al. [36] demonstrated the extraction, hydrolysis, and fractionation of proteins from *R. pulmo*, highlighting proteins as the most abundant class of biocompounds in the jellyfish’s biochemical composition.

Lipid determination. The lipid content was determined gravimetrically using a method adapted from Percy et al. (1981) [113]. Briefly, 100 mg of sample was extracted with a chloroform–methanol mixture (2:1, *v*/*v*) using micro-Soxhlet extraction equipment. The crude lipid extract was subsequently washed with a 0.9% NaCl solution to remove non-lipid contaminants. The solvent was evaporated at 70 °C, and the resulting residue was placed in a desiccator overnight prior to weighing. All measurements were performed in triplicate, and the results are expressed as the mean of three determinations.

Determination of carbohydrates (polysaccharides). Polysaccharides were extracted following the method described by Zhang et al. (2014) [114]. The extraction conditions were optimized as follows: a raw material-to-water ratio of 1:7.5, an extraction temperature of 100 °C, and an extraction time of 4 h. Protein removal from the crude extracts was carried out using papain in combination with Sevag reagent. Polysaccharide fractions were obtained by ethanol precipitation at varying ethanol concentrations of 60%, 70%, and 80%. Notable differences in the physicochemical properties of the precipitated fractions were observed, consistent with findings reported by Migone et al. (2022) and Zhang et al. (2014) [63,114].

#### 4.4.2. Biochemical Composition for Brown Algae *C. barbata*

Determination of protein and total nitrogen. The protein and total nitrogen content of the algae was determined by the Kjeldahl method, using UdK DK6 digester equipment with 127 distillation units and Velp software, Respirosoft™ (Usmate, Italy). First, sulfuric acid mineralization was performed in the presence of catalytic action of mercury and selenium. Then alkalization was performed, and the ammonia was entrained with steam and captured in a boric acid solution, which was subsequently titrated with hydrochloric acid. The results in relation to the amount of seaweed powder used were expressed in percentages [42,115].

Determination of lipids. The lipids were extracted using a slightly modified method from Rohani-Ghadikolalalel et al. (2012) [116]. Extraction of lipids from algal powder samples was performed with dichloroethane by the Soxhlet method for 5 h [111]. After evaporation of the solvent, total lipids were determined gravimetrically on two aliquots of each lipid extract. Results were expressed as percentages, depending on the amount of algal powder used [111,117].

Determination of carbohydrates was made from the aqueous extracts of the brown alga *C. barbata* by the most widely used method, which is based on the DuBois et al. 1956 method, and which was adapted by Albalasmeh et al., 2013 [118]. In this analysis, a solution of 5% phenol in water (*w*/*w*) was prepared immediately prior to the measurement and added to the acid solution containing carbohydrates. Total carbohydrates were estimated spectrophotometrically at a maximum wavelength of 490 nm using the VWR UV-6300 PC double-beam spectrophotometer VWR UV-6300 PC (Leuven, Belgium). There is a strong linear correlation between carbohydrate concentrations and light absorption measured by this method. The results were calculated from a standard glucose calibration curve using the following Equation (2):

y = 0.1009x − 0.0024(2)

where y is the carbohydrate concentration and x is the absorbance. The correlation coefficient is R^2^ = 0.992 [111,118].

Total dietary fiber and its soluble/insoluble fractions were determined using the methods described by Yaich et al. (2015) [119]. The fiber content of the brown algae *C. barbata* was quantified and compared using two different methods: the Englyst procedure (enzymatic–chemical method) and the Prosky method (gravimetric method). Yaich et al. (2015) reported that there is a minimal difference in the total dietary fiber content obtained using these two procedures [119]. However, discrepancies were observed when differentiating between soluble and insoluble fibers. The gravimetric method was found to be simpler to apply.

### 4.5. Jellyfish R. pulmo Physico-Chemical Data for the Collagen Structure

#### 4.5.1. SDS-PAGE Analysis

SDS-PAGE, or sodium dodecyl sulfate–polyacrylamide gel electrophoresis, was used for protein characterization. The laboratory sample was prepared in a phosphate-buffered solution (pH 7.4), and a 10 g mixture was centrifuged for 5 min at room temperature to remove any undissolved material. Equal volumes of the resulting supernatant and buffer solution were mixed and heated at 90 °C for 5 min. The extraction process included dialysis, cell membrane disruption, and protein denaturation at 95 °C using Laemmli reagent. The prepared sample was then loaded onto the gel, which was initially run at 85 V for 30 min, after which the voltage was increased to 95 V until completion. The gel was stained overnight and then decolorized with a solution of acetic acid, menthol, and distilled water. Gel analysis was performed using a calibrated GD-800 densitometer (Bio-Rad Laboratories, Hercules, CA, USA) and analyzed with Quality One software (Bio-Rad 4.6.3). SDS-PAGE analysis was also utilized in similar studies by Addad et al. (2011), James et al. (2023), and De Domenico et al. (2019) [29,34,36].

#### 4.5.2. Circular Dichroism Spectral Analysis

This spectrophotometric analysis was performed to characterize the protein structure. This optical spectroscopy method exploits differential absorption, providing structural information about protein conformations. Using this technique, the secondary structure of proteins can be analyzed in the far UV region (λ between 240 and 170 nm), while the local tertiary structure around aromatic amino acid residues is monitored in the near UV region (λ between 300 and 260 nm). The measurements were conducted with a Jasco J-810 spectropolarimeter. The UV-CD spectrum was obtained using a quartz cuvette with an optical path length of 0.02 cm, a scanning speed of 50 nm/min, and a step size of 0.2 nm, with a response time of 2 s, covering the wavelength range from 250 to 195 nm. The final spectrum represents the average of four consecutive readings at 25 °C. The circular dichroism spectrum of jellyfish collagen from *R. pulmo* showed a minimum at λ = 198 nm and a maximum at λ = 214.5 nm when prepared in a 0.5 M acetic acid solution.

#### 4.5.3. FT-IR Spectroscopy Analysis of Collagen Peptides

Fourier-transform infrared (FT-IR) spectroscopy was performed using a Jasco 4200 FT-IR spectrometer, covering a wavenumber range of λ = 350–7800 cm^−1^, with control provided by JASCO’s exclusive Spectra Manager II software. Collagen samples were dissolved in 0.5 M acetic acid at a 1:1 (*w*/*v*) ratio. The FT-IR spectra were recorded in the infrared (IR) range, with the collagen samples finely pulverized and mixed with KBr. The FT-IR spectra of collagen peptides derived from *R. pulmo* (JPC) were collected in the wavenumber range of 700–4000 cm^−1^.

#### 4.5.4. Amino Acid Analysis of *R. pulmo* Collagen

Amino acid analysis of *R. pulmo* collagen was performed using an HPLC system (626 LC System) equipped with a 717 plus Autosampler, 474 Scanning Fluorescence Detector, and Millennium Chromatography Manager Software (version 3.2, Waters Corporation, Milform, MA, USA). Similar methods were employed by Barzideh et al. (2014) to analyze amino acid compositions in collagen peptides from the ribbon jellyfish (*Chrysaora* sp.) [120]. For the analysis, 0.2 g of lyophilized jellyfish powder was mixed with 5 mL of double-distilled water, 0.75 mL of trifluoroacetic acid, and 50 μL of an internal standard solution (5 M DL-Norleucine in water), and incubated for 20 min. The mixture was then centrifuged at 3500 rpm for 20 min at 4 °C. The supernatant was filtered and dried under nitrogen flow, and the residue was redissolved in 1 mL of double-distilled water. Amino acids were quantified using the AccQ-Tag protocol (Waters, Milford, MA, USA). Each sample was analyzed using a C18 AccQ-Tag column (3.9 × 150 mm), with elution using a phosphate buffer (eluent A) and acetonitrile/water 60:40 (*v*/*v*) (eluent B). The operating conditions included a flow rate of 1 mL/min and a temperature of 37 °C. The fluorescence detector parameters were λ ex = 250 nm, λ em = 395 nm, with an EUFS of 100.

### 4.6. Evaluation of Polyphenol Content

#### 4.6.1. Evaluation of Total Polyphenol Content (TPC) in Marine Resources

Total polyphenol content (TPC) was determined in both collagen extracts from *R. pulmo* (JPC) and in the brown alga extract *C. barbata*, too. For the JPC, the Folin–Ciocalteau colorimetric method was used, with slight modifications. A 100 µL sample of *R. pulmo* was mixed with 500 µL of Folin–Ciocalteau phenolic reagent and 500 µL of sodium carbonate. The mixture was kept for 2h in the dark and then using a spectrophotometer, absorbance was read at 70 nm. For the concentration range of 20–200 µg/mL, gallic acid was used as standard. The calibration curve established by Equation (3):

y = 0.0067x − 0.029(3)

with a correlation coefficient R^2^ = 0.9998.

All samples of JPC were analyzed in triplicate, and the mean value was reported. Results are expressed as gallic acid equivalents (µg GAE/g DW) of lyophilized JPC. The Folin–Ciocalteau method for TPC determination was similarly employed by Leone et al. (2019) and Stabili et al. (2021) [121,122].

For the analysis of polyphenols in *C. barbata*, the same modified Folin–Ciocalteau method was applied. The calibration curve for this analysis was also established by Equation (4):

y = 0.0078x + 0.1842(4)

and the results were calculated accordingly with a correlation coefficient R^2^ = 0.9999. All samples were performed in triplicate and the mean value was reported. The results are expressed as gallic acid equivalent mg GAE/g DW. Cadar et al. evaluated TPC from *C. barbata* [57].

#### 4.6.2. Evaluation of Total Flavonoid Compound (TFC) Content

The colorimetric method was used to obtain the total flavonoid content with quercetin as the standard, following a modified procedure [123,124]. A 1 mg sample of standard quercetin was dissolved in methanol and made up to a final volume of 10 mL (100 ppm). The resulting solution was then diluted to prepare standard solutions at concentrations of 25, 50, 75, and 100 μg/mL. To each quercetin standard solution, 3 mL of methyl alcohol, 0.3 mL of 10% AlCl_3_, and 0.3 mL of 1M potassium acetate were added. Distilled water was added to bring the final volume to 10 mL. For 30 min, the mixtures were incubated at room temperature. Absorbance was measured at a wavelength of 431 nm, with a UV-Vis spectrophotometer. The flavonoid content was determined using the quercetin calibration curve, as provided by Equation (5):

Y = 0.0029x − 0.0123 (5)

with a correlation coefficient R^2^ = 0.9444. The results were expressed as quercetin equivalent mg QE/g fresh seaweed. Cadar (2018) conducted research on the total content of flavonoid compounds in white *C. barbata* [55].

#### 4.6.3. Individual Phenol Content

Individual phenolic compounds in the collagenous extract from *R. pulmo* jellyfish (JPC) and the ethanolic extract from *C. barbata* marine brown algae (ALG) were determined using the method described by Goupy [125]. Detection was performed via high-performance liquid chromatography (HPLC, Agilent 1200 series, Agilent Technologies, Santa Clara, CA, USA), equipped with a quaternary pump, auto-sampler, column compartment set at 35 °C, and a multi-wavelength detector set to 330 nm and 280 nm Retention times and peak areas were automatically monitored and calculated using the Chem 32 integrator (Agilent). For the *C. barbata* extract, the retention times (±standard deviation) of phenolic standards were: pyrogallol acid (0.910 ± 0.025), gallic acid (0.990 ± 0.03), protocatechuic acid (3.130 ± 0.008), 4-amino-benzoic acid (3.455 ± 0.005), chlorogenic acid (3.501 ± 0.015), p-hydroxybenzoic acid (5.933 ± 0.006), vanillic acid (6.919 ± 0.05), caffeic acid (8.281 ± 0.07), ferulic acid (8.865 ± 0.06), benzoic acid (9.468 ± 0.098), ellagic acid (15.303 ± 0.03), and salicylic acid (15.952 ± 0.03), with a 0.05 mg/mL concentration for each. For JPC from *R. pulmo*, the retention times (±standard deviation) of the phenolic acids selected were: gallic acid (3.0278 ± 0.07), caftaric acid (0.4697 ± 0.05), and syringic acid (0.1942 ± 0.03). Individual phenolic acids were identified by comparing the retention times of sample chromatographic peaks with those of authentic standards, using identical HPLC-DAD conditions. Results are expressed as the mean value (±SD) for seaweed samples in mg/100 g fresh weight (f.w.) and as a percentage for the extract of the algae. For JPC, the mean value (±SD) is expressed in mg/100 g fresh weight and as a percentage for the extract of the jellyfish collagen.

### 4.7. Physico-Chemical Characteristics for JPC-ALG Preparations

#### 4.7.1. Organoleptic Characteristics

The newly developed JPC-ALG composites are intended for topical application in wound healing. These composites were prepared in various physical forms, including lyophilized powders, peptide membranes, collagen peptide hydrogels, and collagen peptide films, combined with different concentrations of *C. barbata* brown algae extracts. For each formulation, the color, physical appearance, and organoleptic properties were evaluated. These characteristics varied depending on the specific percentage ratios of JPC to *C. barbata* extract used in the mixtures.

#### 4.7.2. Rheological Characteristics of Preparations

Rheological studies were conducted to evaluate the flow properties of collagen hydrogels combined with seaweed extracts over time, as well as to assess the stability of the pharmaceutical formulations. The rheological behavior was measured at 23 ± 0.1 °C using a Haake VT 550 rheoviscometer equipped with a medium viscosity sensor system and Rheo-Win 4 software (Thermo Fischer Scientific, Waltham, MA, USA). Comparative rheological analyses were performed on JPC-ALG 1 hydrogels containing 10% *Cystoseira barbata* extract and JPC-ALG 2 hydrogels containing 15% *C. barbata* extract. The rheological behavior was evaluated according to Equations (6)–(9), as presented in Table 6.

Based on the results obtained, the rheological model applicable to the prepared soft hydrogels could be determined.

#### 4.7.3. Optical Microscopy Studies for the JPC-ALG Composite

The JPC-ALG composite was studied by optical microscopy on microscope plates with formalin solution. The images were observed and described under an optical microscope: Nikon eclipse imaging system: Nikon Digital Sight DS-FI2, made in Tokyo, Japan.

### 4.8. Antioxidant Activity Was Assessed by DPPH Test and Reducing Power Assay

#### 4.8.1. DPPH Test

The method is based on the scavenging effect on the free radical α-diphenyl-β-picrylhydrazyl (DPPH) and was measured according to the procedure described by Brand et al. (1995) [126]. DPPH is a stable purple free radical, which, upon reaction with antioxidants present in the sample, is reduced and undergoes a color change from purple to yellow. Briefly, a 1 M DPPH solution in ethanol was prepared, from which 4 mL was mixed with 1.5 mL of the JPL extract at various concentrations. For 30 min, the mixture was incubated at room temperature. Then with a UV-VIS spectrophotometer, the absorbance at 517 nm was read. The DPPH activity was calculated as a percentage with Equation (10):

(10) $$\mathrm{DPPH}\;(\%)=\frac{A_{control}-A_{sample}}{A_{control}}\times 100$$

where A_control_ is the absorbance of the control, and A_sample_ is the absorbance of the tested extract. The positive control was ascorbic acid. The procedure was applied to both the *C. barbata* algae extracts and the newly developed pharmaceutical preparation JPC-ALG. Concentrations of JPC collagen extracts, brown algae extracts, and the JPC-ALG formulations that resulted in IC_50_ values (defined as the concentration of the sample capable of inhibiting 50% of the total DPPH radicals) were determined from the corresponding graphs using nonlinear regression analysis. Results are expressed as the mean ± SD of three parallel measurements.

#### 4.8.2. Reducing Power Assay

The reducing power assay is widely employed to evaluate the antioxidant activity of polyphenols. Reducing power is generally attributed to the presence of reductones, which exert antioxidant action by breaking free radical chains and donating hydrogen atoms, as shown by Hsu et al. (2003) [127]. Cadar et al. (2019) applied a slightly modified version of the method originally described by Oyaizu (1986) [57,128]. In this assay, 1 mL of JPC extract from *R. pulmo* was mixed with 2 mL of 0.3 M phosphate buffer (pH 6.6) and 2 mL of 1% potassium ferricyanide. The mixture was incubated at 55 °C for 30 min, after which 2 mL of 10% trichloroacetic acid was added. A 2 mL aliquot of the incubation mixture was then combined with 3 mL of distilled water and 1 mL of 0.1% ferric chloride in test tubes. After 10 min, the absorbance was read at 700 nm wavelength using a UV-Vis spectrophotometer. Ascorbic acid (concentration 200 μg/mL) was used as standard. The same method was applied to the hydroalcoholic extract of *C. barbata* (ALG) and to the newly developed JPC-ALG formulations. Results are expressed as the mean ± SD of three parallel measurements.

### 4.9. Antimicrobial Activity

For antibacterial activity evaluation, two Gram-positive bacterial species, *Staphylococcus aureus* (ATCC 25923) and *Staphylococcus epidermidis* (ATCC 12228), and four Gram-negative species, *Klebsiella pneumoniae* (ATCC 13883), *Pseudomonas aeruginosa* (ATCC 27853), *Proteus mirabilis* (ATCC 25933), and *Escherichia coli* (ATCC 25322), were used. The bacterial strains and culture media were provided by the Veterinary and Food Safety Directorate in Constanța, Romania. Strains were cultured on an agar medium at 37 °C. The agar medium was composed of 5 g peptone, 3 g beef extract, 5 g NaCl, and 20 g agar dissolved in distilled water (pH 7.2–7.4). Bacterial suspensions were spread evenly across the plates to obtain uniform lawn cultures [129,130,131]. The antimicrobial activities of the new JPC-ALG preparations, the 10%, 15%, and 20% ethanolic hydroalcoholic extracts of brown algae *C. barbata*, and the JPC extracts from *R. pulmo* were evaluated using the agar well diffusion method with slight modifications. Antimicrobial efficacy was assessed by measuring the diameter of the inhibition zones according to the Kirby–Bauer diffusimetric method, and the results are reported as the mean of three independent experiments [130]. A chloramphenicol solution (5 mg/mL) served as the positive control, while 85% ethanol was used as the negative control. The minimum inhibitory concentration (MIC) was determined by evaluating the smallest concentration of extract that produced a visible zone of inhibition after incubation, as reported by Tajbakhsh et al. (2011) [129]. The plates were incubated at 37 °C for 24 h, and the diameters (mm) of the inhibition zones were recorded as an index of antibacterial activity, as recommended by Li et al. (2018) [130]. MIC measurements were carried out for the ethanolic extracts of *C. barbata* (ALG), the JPC hydrolysates, and the new JPC-ALG composites.

### 4.10. Biological Investigation

#### 4.10.1. Cell Viability

Cell viability assays were performed on both fibroblasts and keratinocytes. BALB/3T3 fibroblast clone A31 (CCL-163) cells were purchased from ATCC (New York, NY, USA), and HaCaT keratinocyte cells were obtained from Cell Systems GmbH (Eppelheim, Germany). Cells were cultured and maintained in a CO_2_ incubator at 37 °C with 5% CO_2_ and subcultured at 80–90% confluence. The culture medium used was DMEM (Dulbecco’s Modified Eagle Medium) supplemented with 2 mM L-glutamine, 1% penicillin/streptomycin, and 10% bovine calf intestinal serum (Merck, Darmstadt, Germany). Cell monolayers were rinsed with phosphate-buffered saline (PBS) and detached using trypsin-EDTA prior to resuspension in a fresh medium, as described by Migone et al. (2022) [63].

For the viability assay, BALB/3T3 cells were seeded at densities of 1 × 10^4^ and 2.5 × 10^3^ cells per well, while HaCaT cells were seeded at 3 × 10^4^ and 2 × 10^4^ cells per well in 96-well plates, depending on whether viability was assessed at 24 or 48 h, respectively. Cells were incubated at 37 °C with 5% CO_2_ for 24 h to allow proliferation before treatment. The culture medium was replaced with fresh medium containing the tested material, fully dissolved in DMEM at concentrations ranging from 0.01 to 2 mg/mL. After 24 or 48 h of incubation, the medium was replaced with fresh medium containing 10% WST-1 reagent. Cells were then incubated for an additional 4 h in 5% CO_2_ at 37 °C. Finally, the formazan product absorbance was measured at 450 nm. The reference wavelength was 655 nm, using a multimode microplate reader (BioTek 800/TS, Thermo Scientific, Waltham, MA, USA).

#### 4.10.2. Scratch Test on BALB/3T3 Cells and HaCat Cells

The in vitro wound scratch assay was conducted with the support of the County Clinical Emergency Hospital, Constanța, Romania. The experimental procedure followed the protocols described by Migone et al. (2022) and Fabiano et al. (2021) [63,132]. BALB/3T3 fibroblasts were seeded in 12-well plates in DMEM supplemented with 1% calf serum. The density of BALB/3T3 fibroblasts was 1.25 × 105 cell per well. Full confluence was obtained after 24 h of incubation in a 5% CO_2_ atmosphere at 37 °C. Similarly, HaCaT keratinocytes were seeded at the same density in DMEM (supplemented with 1% fetal bovine serum) and reached complete confluence after 48 h under identical conditions.

Upon reaching confluence, the monolayers were scratched using a sterile 200 µL pipette tip (200). Wells were immediately washed three times with PBS to remove cellular debris and non-adherent cells. Subsequently, 2 mL of the test sample (final concentration 0.125 mg/mL per well) was added. Culture medium alone was used as a control. Micrographic images were captured immediately after scratching (time zero), and subsequently at 4, 24, and 48 h using a Nikon Eclipse Ts2R inverted microscope equipped with a 4× objective.

The scratch closure (confluence rate) was quantified by analyzing the acquired images using ImageJ software 1.54 version (NIH, USA) [133]. The percentage of wound closure over time was calculated according to Equation (11):

(11) $$Confluency\;rate\;\left(\%\right)=\frac{Area\;T_{o}-Area\;T}{Area\;T_{o}}\times 100$$

where Area To is the area at time zero and Area T is the area at each endpoint.

Cell migration activity was expressed as a percentage. It represents the gap between the surface area obtained with treatment versus the total surface area of the cell-free region immediately after scratching.

### 4.11. Statistical Methods

Results were studied using SPSS 16.0 parametric tests. One-way ANOVA was used to correlate data on the chemical composition of the marine material groups (JPC from *R. pulmo* or ALG from *C. barbata*). All values obtained from experiments are presented as mean ± SD of three (or more) parallel measurements. When differences were found, Duncan’s multiple comparison test and the paired Student’s *t*-test were used. Data were processed as means ± SEM, and the level of statistical significance was set at * *p* < 0.05 for most statistically processed analyses and ** *p* < 0.01 for biochemical analyses performed on cell cultures.

## 5. Conclusions

The wound-healing process constantly requires innovative treatments to ensure effective healing, relieve patients’ pain, and reduce healthcare costs. The use of natural products in wound-healing treatments has begun to be considered in order to achieve products with potential that are within the reach of users. This study presents some notable innovations in marine-derived materials for wound healing. Unlike many previous studies investigating collagen or algal extracts in isolation, we have developed a synergistic composite hydrogel (JPC-ALG) that combines *R. pulmo* collagen peptides with *C. barbata* brown algae extracts. This is the first report to evaluate this specific combination from materials harvested from the Romanian Black Sea coast, highlighting not only their physicochemical properties but also their complementary bioactive profiles, including enhanced effects of activities such as antioxidant, antimicrobial, and cell migration. The study offers a novel formulation approach using a stepwise integration process that preserves the triple-helix structure of collagen while capitalizing on the rich phenolic and polysaccharide content of brown algae. In addition, our work integrates comprehensive in vitro assessments (fibroblast and keratinocyte scratch assays, cytocompatibility, antioxidant, and antimicrobial assays) into a single, coherent bioproduct development workflow. This marine-derived composite also stands out due to its favorable rheological behavior, which supports its application in topical biomedical formulations. Previous research has largely focused on collagen from terrestrial sources or algal gels with limited functions. In contrast, our formulation demonstrates a functional synergy that enhances both mechanical properties and biological performance, making it a promising platform for next-generation wound dressings. The present study, designed as a preliminary investigation, constitutes the first phase of a larger research project aimed at the development of marine-derived biomaterials for wound care. This work provides a fundamental step towards the clinical application of marine-derived biocomposites for wound-healing applications, highlighting their ability to stimulate cell proliferation, migration, and antimicrobial defense mechanisms essential for efficient skin regeneration. Despite these promising results, we have to note the occurrence of several limitations that have emerged during the course of the research. First, the lack of in vivo animal testing prevents a complete understanding of the formulation’s pharmacokinetics, biodegradation, and tissue integration behavior under physiological conditions. Second, the scalability and reproducibility of collagen and algal extract preparation processes need to be optimized and validated for industrial production. Third, long-term storage stability and sterility of the final composite remain to be evaluated under clinical conditions. Future research will address all these limitations in turn by addressing animal injury models, optimizing scale-up, and developing sterile, storage-stable formulations. We emphasize that this study requires more future in vivo investigations, which are currently being organized legislatively and administratively and will involve in vivo animal models of wound treatment. However, further studies are needed to address challenges related to the structures, extraction processes, and potential cytotoxicity of these extracts, which may hinder the development of novel therapeutics. More studies are needed to evaluate the composite’s biocompatibility, safety, degradation behavior, and efficacy in promoting wound closure and tissue regeneration in vivo. Further studies are needed to validate the applicability and support further clinical development of this biomaterial.

In addition, strategic initiatives are needed at the European level to organize sustainable harvesting of jellyfish and brown algae, transforming them into valuable natural bioactive compounds with biomedical applications.

In conclusion, jellyfish and brown algae, through their bioactive compounds, represent a promising direction for the development of new medical therapies. These include the manufacture of biomaterials based on jellyfish collagen peptides, treatments based on brown algal polysaccharides, new pharmaceutical formulations for topical applications, and advances in tissue engineering.

## Figures and Tables

**Table 1:** The characteristics of collagen peptides hydrogels and hydroalcoholic algal extract.

Characteristics	Hydrogels Extract JPC from *R. pulmo*		Algal Hydroalcoholic Extract ALG *C. barbata*	
	with 10% Pepsin	References	*C. barbata*	References
Moisture % (DW)	15.1 ± 0.1	-	12.6 ± 0.33	12.27 ± 0.42 [49]
Ash 600–800 °C % (DW)	0.55 ± 0.1	-	17.28 ± 0.88	18.63 ± 1.73 [49]
Proteins % (DW)	60.48 ± 1.72 23.59 ± 1.89 B; 32 ± 1.19 OA; 17.56 ± 1.98 G	61.8 [46] 6 W; 8.7–13.7 B; [47] 27 OA; 18 G [47]	20.98 ± 0.65	18.13 ± 2.11 [49]
Collagen content % (DW)	57.1 ± 0.6	56.3 [36]	-	-
Lipid % (DW)	4.9 ± 0.81 W; 1.95 ± 0.3 G	2.3 W; [47] 4.0 ± 0.1 W; 0.8 OA; 1.2 G; [47]	6.28 ± 0.58	1.63 ± 0.54 [49]
Carbohydrates % (DW)	0.59 ± 1.25 W; 0.25 ± 0.65 G	-	60.25 ± 1.56	61.95 ± 1.06 [49]
Total dietary fiber % (DW)	-	-	59.26 ± 1.05	61.075 ± 1.66 [49]
Insoluble fiber % (DW)	-	-	28.22 ± 1.42	30.62 ± 1.26 [49]
Soluble fiber % (DW)	-	-	31.04 ± 1.03	30.45 ± 1.33 [49]

Data are percentages of dry weight (DW) in the whole (W) or different body parts (B, bell; OA, oral arms: G, gonads). ±SD for *n* = 3.

**Table 2:** Amino acid content identified in *R. pulmo*.

Amino Acids	*R. pulmo* from Black Sea Coast Residues/1000 Residues	*R. pulmo* from Goa Coast India [34] %	*R. pulmo* from Mediterranean Sea [46] mg/100 g
Tissue	Whole body	Whole body	Whole body
Essential amino acids (EAAs)			
Arginine (*Arg*)	6.2	5.63	1.8
Cystine (*Cys*)	1.2	-	1.2
Glutamic acid (*Glu*)	15.2	13.46	13.7
Glycine (*Gly*)	33.4	29.34	4.8
Histidine (*His*)	0.6	-	5.0
Isoleucine (*Ile*)	-	-	4.9
Leucine (*Leu*)	8.6	6.35	8.2
Lysine (*Lys*)	6.3	4.62	6.2
Methionine (*Met*)	-	-	4.1
Proline (*Pro*)	3.9	2.97	3.5
Hydroxiproline (*Hyp*)	3.65	4.82	-
Phenylalanine (*Phe*)	-	-	8.4
Threonine (*Thr*)	5.25	3.18	4.5
Triptophan (*Trp*)	2.8	4.72	-
Tyrosine (*Tyr*)	3.90	1.77	6.8
Valine (*Val*)	4.9	2.8	4.4
Non-essential aminoacids (NEAAs)			
Alanine (*Ala*)	6.9	10.38	3.5
Aspartic acid (*Asp*)	6.65	10.91	2.9
Serine (*Ser*)	1.7	-	6.0

**Table 3:** Individual polyphenolic compounds in *C. barbata* and *R. pulmo* extracts from the Black Sea.

Type of Acid	Mean Value for Extract ALG ± SD mg/100 g f.w.	Percentage for Extract ALG%	Mean Value for JPC ± SD mg/100 g f.w.	Percentage for JPC%
Pyrogallol Acid	4.2 ± 0.05	1.36	-	-
Gallic Acid	3.5 ± 0.03	1.13	5.84 ± 0.02	88.75
Protocatechuic Acid	7.12 ± 0.01	2.3	-	-
4-Amino-benzoic Acid	5.2 ± 0.09	1.68	-	-
Chlorogenic Acid	5.3 ± 0.05	1.71	-	-
p-Hydroxy-benzoic Acid	26.9 ± 0.06	8.70	-	-
Vanillic Acid	99.5 ± 0.08	32.18	-	-
Caffeic Acid	21.2 ± 0.06	6.86	-	-
Caftaric Acid	-	-	0.24 ± 0.01	3.65
Feluric Acid	54.5 ± 0.01	17.62	-	-
Benzoic Acid	65.7 ± 0.06	21.25	-	-
Ellagic Acid	5.6 ± 0.02	1.81	-	-
Salicylic Acid	10.5 ± 0.03	3.4	-	-
Syringic Acid	-	-	0.50 ± 0.009	7.60

±SD for 3 replicates.

**Table 4:** Biocomposites from marine resources for wound healing.

Appearance	Color	Appearance
Collagen peptides from jellyfish *R pulmo*	white	powder
Lamellar film from jellyfish *R. pulmo*	white	showing porosity
Collagen peptide hydrogel from *R. pulmo* with hydroalcoholic extract of *C. barbata* 5%	yellowish white	gelatins viscous
*R. pulmo collagen* peptide hydrogel with hydroalcoholic extract of *C. barbata* 10%	white yellow dark	gelatins viscous
*R. pulmo*-collagenic peptide films with hydroalcoholic extract of *C. barbata* 5%	yellowish white	porous composite material
Collagen peptide hydrogel of *R. pulmo* with hydroalcoholic extract of *C. barbata* 10%	white yellow dark	viscous composite material
*R. pulmo collagen* peptide films with hydroalcoholic extract of *C. barbata* 15%	brownish white	porous composite material

**Table 5:** MIC of extracts from the Romanian Black Sea coast. (*n* = 3).

Type of Bacteria	MIC (µg/mL)		
	JPC *R. pulmo*	ALG *C. barbata*	JPC-ALG
*Escherichia coli*	75 ± 0.3	75 ± 0.2	75 ± 0.3
*Pseudomonas aeruginosa*	50 ± 0.6	50 ± 0.5	50 ± 0.4
*Proteus mirabilis*	25 ± 0.5	25 ± 0.4	25 ± 0.5
*Klebsiella pneumonia*	50 ± 0.3	75 ± 0.1	75 ± 0.2
*Staphylococcus aureus*	75 ± 0.4	>100 ± 0.1	>100 ± 0.1
*Streptococcus epidermidis*	50 ± 0.5	50 ± 0.4	50 ± 0.4

**Table 6:** Rheological parameters and equations.

Viscosity ɳ (cP) Depending on Shear Speed D (s^−1^)	Shear Speed D (s^−1^) in Correlation with the Selected Rotation Speed ω (rpm)	Shear Speed D (s^−1^) Depending on Shear Stress τ (Pa)	Shear Stress τ (Pa) Depending on Viscosity ɳ (cP) and SHEAR Speed D (s^−1^)
ɳ = f(D) (6)	D = ω * R (7)	D = f(τ) (8)	τ = ɳ * D (9)

## Data Availability

The raw data supporting the conclusions of this article will be made available by the authors on request.

## Abbreviations

JPC	Jellyfish Collagen Peptides
ALG	Alga (brown algae *C. barbata*)
JPC-ALG	Composite Jellyfish Peptides-Brown Algae
SDS-PAGE	Sodium Dodecyl Sulfate–Polyacrylamide Gel Electrophoresis
FT-IR	Fourier-Transform Infrared Spectroscopy
ASC	Acid Solubil Collagen process
PSC	Pepsin Soluble Collagen
TPC	Total phenolic content
TFC	Total flavonoid content
DPPH	2,2-diphenyl-1-picrylhydrazyl
MIC	Minimal inhibitory concentration
BALB/3T3	A fibroblast cell line
HaCaT	Keratinocyte cell line
ECM	Extracellular Matrix
ROS	Reactive Oxygen Species
TGF-α	Transforming Growth Factor-Alfa
TGF-β	Transforming Growth Factor-Beta
FGF	Fibroblast Growth Factor
PDGF	Platelet-Derived Growth Factor
VEGF	Vascular Endothelial Growth Factor
